# Genome-wide identification of the *TGA* genes in common bean (*Phaseolus vulgaris*) and revealing their functions in response to *Fusarium oxysporum* f. sp. *phaseoli infection*

**DOI:** 10.3389/fgene.2023.1137634

**Published:** 2023-01-20

**Authors:** Yu Liu, Yuning Huang, Zhao Li, Ming Feng, Weide Ge, Chao Zhong, Renfeng Xue

**Affiliations:** ^1^ College of Agronomy, Shenyang Agricultural University, Shenyang, China; ^2^ Crop Research Institute, Liaoning Academy of Agricultural Sciences, Shenyang, Liaoning, China; ^3^ Liaoning Provincial Key Laboratory of Miscellaneous Grain Germplasm Innovation and Genetic Breeding, Shenyang, China

**Keywords:** Common bean, TGA gene family, Gene family, *Fusarium oxysporum* f*.* sp. *Phaseoli*, Fusarium wilt

## Abstract

Fusarium wilt, which affects common bean all across the world, is caused by *Fusarium oxysporum* f. sp. *Phaseoli* (*Fop*). It is necessary to have functional genes in response to *Fop* infection because they might be used to manage disease. As a crucial regulator, *TGA*-binding transcription factor (*TGA*) is engaged in the defense mechanism of plants against pathogens. The role of *TGA* regulators in common bean in response to *Fop* infection, however, has not been documented. Hence, we performed genome-wide identified and characterized eight *TGA* genes in common bean. In this study, eight *PvTGA* genes were distributed on six chromosomes and classified into four subgroups. The *PvTGA* genes have the same conserved bZIP and DOG1 domains, but there are specific sequence structures in different *PvTGAs*. Phylogenetic and synteny analysis explained that *PvTGA* gene has a close genetic relationship with legume *TGAs* and that *PvTGA03* and *PvTGA05* may play an important role in evolution. Transcriptome data explained that expression levels of *PvTGA* genes showed diversity in different tissues. After *Fop* inoculation, the expression levels of *PvTGA0*3 and *PvTGA07* were significantly different between resistant and susceptible genotypes. Under SA treatment, the expression levels of *PvTGA03*, *PvTGA04*, *PvTGA06*, *PvTGA07* and *PvTGA08* were significantly different. These results imply that *PvTGA03* and *PvTGA07* play key roles in SA-mediated resistance to Fusarium wilt. Together, these findings advance knowledge of the *PvTGA* gene family in common bean and will help future studies aimed at reducing Fusarium wilt.

## 1 Introduction

Common bean (*Phaseolus vulgaris* L.) is an economically important leguminous crop grown worldwide. It is an important source of fiber, proteins, vitamins, and essential micronutrients for human nutrition (Blair et al., 2013; Blair et al., 2016; Suárez-Martínez et al., 2016). The planting area of common bean is second only to soybean and peanut. Common bean is widely cultivated as an important food source in the world, especially in parts of Africa and the South America (Pérez-Vega et al., 2010; Schmutz et al., 2014). The production of common bean is limited by a variety of environmental factors like biotic and abiotic stress (Schwartz et al., 2005). A major fungus disease of common bean is Fusarium wilt, a soil-borne disease that affects a wide variety of crops globally. It is caused by *Fusarium oxysporum* f. sp. *phaseoli* (*Fop*), and has been found and identified in most bean-growing regions of the world. Wet environments or densely planted lands with inadequate crop rotation are especially susceptible to Fusarium wilt (Buruchara and Camacho, 2000). At present, the most effective and environmentally friendly way to control the disease is to discover the resistant genes in common bean, clarify their disease-resistant mechanisms, and apply them to common bean.

Plants have developed a number of barrier defense strategies to shield themselves against invasive pathogens like bacteria, fungi, viruses, and oomycetes (Jones and Dangl, 2006; Spoel and Dong, 2012; Bigeard et al., 2015). Plants activate local defenses that trigger a secondary immune response known as systemic acquired resistance (SAR). SAR provides long-lasting immunity to the distal uninfected tissues by activating various pathogenesis-related genes (Fu and Dong, 2013; Zhou and Zhang, 2020). To date, several molecules have been proposed as mobile signals leading to SAR (Dempsey and Klessig, 2012; Klessig et al., 2018). Salicylic acid (SA) is a phytohormone which plays a role in numerous plant physiological processes in including abiotic or biotic stress regulation. For the initiation of the SAR response and the expression of pathogenesis-related genes (PRs), SA is an essential signaling molecule (Malamy et al., 1990; An and Mou, 2011). The key genes in the synthesis and regulation pathway of salicylic acid have been proved to play an important role in the interaction between common bean and *Fusarium oxysporum* (Xue et al., 2014; Xue et al., 2021). Therefore, it is of great significance to explore the key genes of common bean resistance to Fusarium wilt mediated by salicylic acid and to clarify their functions for analyzing the molecular mechanism of common bean and *Fusarium oxysporum* interaction.

Transcription Factors (TF) are proteins that can regulate the transcription of genes by binding to their specific *cis*-regulatory sequences in the promoter region. TF are crucial in a variety of physiological functions, including metabolic balance, growth, development, and response to adversity stress (Baillo et al., 2019). *TGA* transcription factors (TGACG motif-binding factor) are members of the Basic Leucine Zipper (bZIP) transcription factor family, which is one of the biggest and most significant TF families. *TGA* TFs interact with the SA-receptor NPR protein and bind specifically the activation sequence-1 (as-1) motif in the promoter region of *PR* genes during plants immune response after pathogen attack in *Arabidopsis thaliana* (Rochon et al., 2006)**.** The first *TGA* gene cloned in plants was *TGA1a,* and serves as a crucial point of reference for identifying *TGA* gene family (Katagiri et al., 1989). More *TGA* transcription factors were subsequently found in diverse plants (Jakoby et al., 2002; Idrovo Espín et al., 2012). A total of 10 *TGA* transcription factor genes were identified genome-wide in *Arabidopsis* (Gatz, 2013). Based on their sequence similarities, the *TGAs* can be classified into five subgroups (I-V). The two *TGAs* that most closely resemble tobacco *TGA1a* are found in Group I: *TGA1* and *TGA4*. *TGA2*, *TGA5*, and *TGA6* make up Group II; they are connected and have functional redundancy. *TGA3* and *TGA7* make up Group III, *TGA9* and *TGA10* make up Group IV, and *TGA8* is the solitary member of Group V. Among these identified *TGAs*, *TGA1-TGA7* has been proven to interact with key resistance-related genes such as *NPR1*, and participate in multiple signal regulation pathways in plants to improve plant biotic and abiotic stress resistance (Johnson et al., 2001; Choi et al., 2004; Thurow et al., 2005; Jiang et al., 2021; Liu et al., 2022). In addition to *Arabidopsis* and tobacco, *TGA* has been identified in a variety of plants related to disease resistance, such as the *TGA* gene in soybean responds to mosaic virus, kiwifruit *TGAs* plays a role in the process of *Pseudomonas syringae pv. Actinidiae* (*Psa*) infection (Jiang et al., 2021; Liu et al., 2022). In *Brachypodium distachyon*, the *TGA*-promoted transcription of SA-inducible PR1 is orchestrated by the activator BdNPR2 and the repressor BdNPR1 to enforce immune resistance (Shimizu et al., 2022). However, little is known about *TGA* gene functions in common bean. At the same time, it is not clear how the *TGA* family plays a regulatory role in the process of common bean resistance. Therefore, the objectives of this study were to identify members of the *TGA* gene family in the *Phaseolus vulgaris* L. genome. The phylogenetic relationships, structural features, chromosomal locations, *cis*-elements, and expression patterns of *PvTGA* genes under *Fop* stress were described and analyzed in order to further investigate the function of the *PvTGA* gene family in resistance-related functions and the regulatory mechanisms in common bean. The findings offer a theoretical framework for understanding the role of *TGA* genes in common bean, which may be applied to help create common bean cultivars resistant to Fusarium wilt.

## 2 Materials and methods

### 2.1 Plant material and *Fop* isolates

Common bean genotypes BRB130 (Fusarium wilt susceptible) and CAAS260205 (Fusarium wilt resistant) were obtained from the Institute of Crop Sciences of the Chinese Academy of Agricultural Sciences (CAAS) in Beijing, China (Xue et al., 2017; Xue et al., 2021). An aggressive *F. oxysporum* f. sp. *phaseoli* (*Fop*) isolate, FOP-DM01 was used for the experiments as further described by Xue et al. (2012). BRB130 and CAAS260205 plants were inoculated at the fully expanded, “cotyledonary” leaf, seedling development stage using seedlings that had been cultivated in a greenhouse for 10 days using previous described methods (Xue et al., 2017). To explore responses to SA, Fusarium wilt susceptible genotype BRB130 leaves were sprayed for five consecutive days with 2 mM salicylic acid (SA) diluted in Tween 20 (0.02% v/v) using previous described methods (Xue et al., 2014). The plants were grown in a greenhouse with a temperature range of about 22°C to 28°C. For a total of 14 days, all treatments were grown in the same greenhouse with natural sunshine and additional illumination. In the case of the *Fop* therapy, root tissues were taken at 0, 24, 48, 72, 96, and 120 h post inoculation (hpi), with an additional 6 h collection for the SA treatment. All obtained root tissues were stored at −80°C.

### 2.2 Identification of members of the *TGA* gene family in common bean

To identify *TGA* genes in common bean, 10 *TGA* gene sequences (*AtTGA1* (*AT5G65210*), *AtTGA2* (*AT5G06950*), *AtTGA3* (*AT1G22070*), *AtTGA4* (*AT5G10030*), *ATTGA5* (*AT5G06960*), *AtTGA6* (*AT3G12250*), *AtTGA7* (*AT1G77920*), *AtTGA8* (*AT1G68640*), *AtTGA9* (*AT1G08320*) and *AtTGA10* (*AT5G06839*)) in *Arabidopsis* were selected to search for candidate *TGA* genes in common bean. The amino acid sequence of the AtTGA proteins in *Arabidopsis* was used as queries for a BLAST search for TGAs in the common bean protein database (*Phaseolus vulgaris* v2.1) (https://phytozome-next.jgi.doe.gov/), and the newly searched proteins whose E-value <10^–5^ were then used to perform a BLAST search again to determine all the orthologs. All the discovered proteins were submitted to the databases CDD (https://www.ncbi.nlm.nih.gov/cdd), Pfam (http://pfam.xfam.org/), and SMART (http://smart.embl-heidelberg.de/) to check if they included full bZIP and DOG1 domains in order to further confirm TGA proteins. ExPASy (https://web.expasy.org/protparam/) was used to determine the theoretical isoelectric point (pI) and molecular weight (MW) for the retrieved TGA protein sequences. WoLF PSORT (https://www.genscript.com/wolf-psort.html) online software was used for subcellular localization.

### 2.3 Conserved domains and gene structure analysis of *TGA* gene in common bean

To classify *PvTGAs*, gene cluster analysis was conduction with the common bean and *Arabidopsis* TGA protein using the maximum-likelihood (ML) method. The MEME program (http://meme-suite.org/) was used to detect conserved motifs of the TGA proteins with the following parameters: the width of the motif ranged from 6 to 50 amino acids, with a maximum of 20 (Bailey et al., 2006). *PvTGA* gene structure and conserved domain map were drawn using the Gene Structure View program in TBtools based on the download common bean genome annotation files (*Phaseolus vulgaris* v2.1) (Chen et al., 2019).

### 2.4 Phylogenetic and gene collinearity analysis of *TGA* gene

To further understand the phylogenetic relationship of PvTGA proteins and other plant species, the phylogenetic tree was constructed. TGA proteins from *Arabidopsis thaliana* (Cheng et al., 2017), peanut (*Arachis hypogaea*) (Bertioli et al., 2019), soybean (*Glycine max*) (Li et al., 2019), grape (*Vitis vinifera*) (The French–Italian Public Consortium for Grapevine Genome Characterization, 2007), alfalfa (*Medicago sativa*) (Tang et al., 2014), chickpea (*Cicer arietinum*) (Varshney et al., 2013), rice (*Oryza sativa, Oryza sativa* Japonica Group, www.ncbi.nlm.nih.gov), maize (*Zea mays*, ZmB73_RefGen_v4, www.maizegdb.org) and Sorghum (*Sorghum bicolor*) (McCormick et al., 2018) were obtained in the same way as common bean. The maximum-likelihood (ML) tree was constructed after the TGA protein sequences were aligned in MEGA-X (https://www.megasoftware.net/) using the MUSCLE method (Kumar et al., 2018).

Gene collinearity and genes involved in duplication were examined using the MCScanX (Wang et al., 2012). Analysis was done on the PEBP homologs between common bean and other plant species. We calculated the homologous genes’ non-synonymous to synonymous mutation rate (Ka/Ks) to look into selection pressure. To evaluate selection pressure, the Ka/Ks Calculator 2.0 program was used to calculate the Ka (non-synonymous substitution rate) and Ks (synonymous substitution rate) values of repeated genes (Wang et al., 2010).

### 2.5 *Cis*-element analysis in promoters

The 2,000 bp upstream area of the start codon (ATG) was extracted and submitted the sequences to the PlantCARE (http://bioinformatics.psb.ugent.be/webtools/plantcare/html/) database to predict the *cis*-elements in the promoter of *PvTGA* genes. The distribution of *Cis*-elements of *PvTGA* genes was displayed by TBtools (Chen et al., 2019).

### 2.6 Tissue-specific and post-inoculation expression of *TGA* genes based on RNA-seq

The transcriptome data of gene expression in 11 different tissues during the whole growth period of common bean species are from Phytozome 13 genome database including root_10, nodules, root_19, young pods, stem_10, stem_19, green mature buds, leaves, young triloliates, flower buds, and flower. The expression levels *in silico* were measured using FPKM (expected number of fragments per kilobase of transcript sequence per millions of base pairs sequenced). Using gene expression data in 11 tissues of common bean, the FPKM value of the extracted eight of common bean *TGA* gene was transformed with log_2_^FPKM^. The heatmap program on TBtools platform was used to cluster and draw the heat map.

### 2.7 Quantitative RT-PCR analysis for gene expression

All root tissues were selected for RNA extraction. Using the Plant Total RNA Extraction Kit (Tiangen Biotech, Beijing, China), total plant RNA was extracted. The Reverse Transcription Kit PrimeScriptTM RT Kit (TaKaRa, Japan) was used to create the reagent cDNA, and the process was based on its guidelines. PrimerBlast (https://www.ncbi.nlm.nih.gov/tools/primer-blast/) was used to searching primers for identifying *TGA* genes with differential expression. The internal control was the *Actin11* gene. The SYBR Premix Ex TaqII kit (TliRNaseH Plus) (TaKaRa, Japan) was used to build the reaction system for gene expression analysis, and the ABI7500 was used to detect fluorescence quantitative reactions (Applied Biosystems, United States). The 2^−ΔΔCT^ approach was used to calculate the relative expression analysis (Livak and Schmittgen, 2001).

## 3 Result

### 3.1 Identification and distribution on chromosomes of PvTGA protein in common bean

**FIGURE 1:**
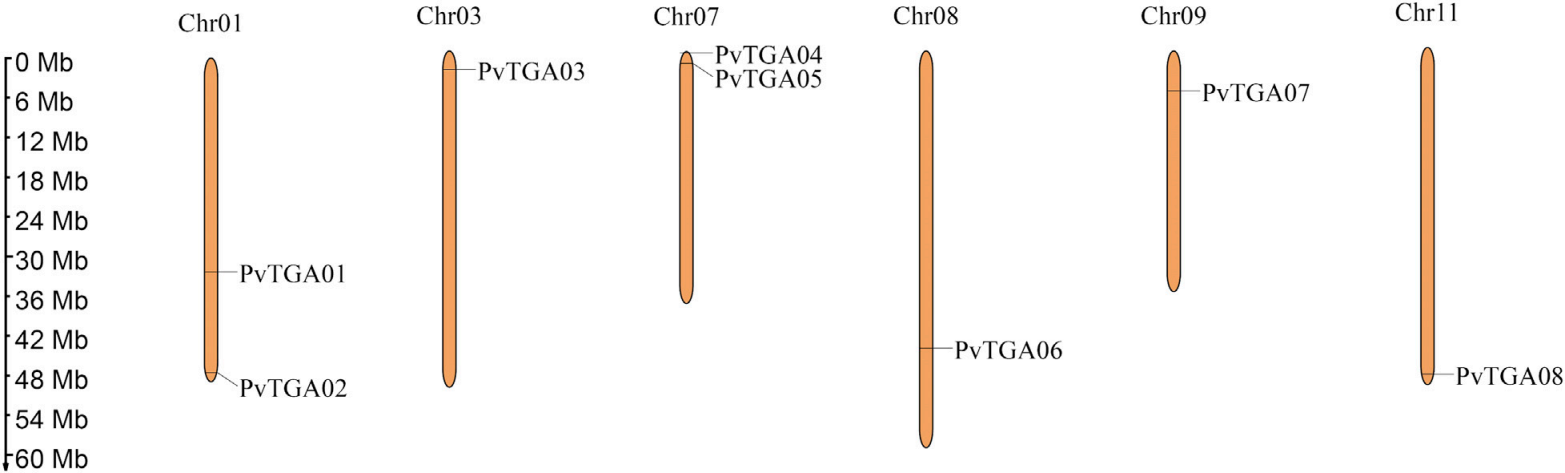
Distribution of *TGA* genes on *P. vulgaris* chromosome. Chromosome size is indicated by its length. The scale on the left is shown in megabases (Mb).

In this study, 10 *AtTGAs* in *Arabidopsis thaliana* were used as a query to identify the *TGA* genes in the common bean genome, a total of eight putative *PvTGA* genes were identified. All gene sequences included complete bZIP and DOG1 domains, according to the findings of BLAST analysis. The common bean genome ultimately included eight *PvTGA* genes, and the relevant genes were renamed *Pv*TGA01–*Pv*TGA08 in accordance with the order of their distribution on different chromosomes. Eight *PvTGA* genes are located on the six chromosomes of common bean (Figure 1). The full length of the candidate TGA protein sequence, the corresponding gene at the chromosomal location, the molecular weight (MW) of the proteins encoded, subcellular localization and the isoelectric point (pI) were summarized (Table 1). The full length of the eight protein sequences ranges from 351 to 467. The molecular weight (kDa) and theoretical isoelectric points (pI) of putative TGA proteins ranged from 39.72 (PvTGA07) to 51.71 (PvTGA03) kDA and 6.03 (PvTGA05) to 8.53 (PvTGA01), respectively. Additionally, predictions of protein subcellular localization revealed that *AcTGA* genes were primarily found in cell nuclei.

**TABLE 1:** The genomic and biochemical information for *TGA* genes identified in common bean.

Gene name	Locus ID	Chromosomal location	mRNA (bp)	Protein (aa)	LOC	MWb (kDa)	PI
*PvTGA07*	Phvul.009G026900.1.v2.1	Chr09:6317481.6321181	1701	351	nucl:14	39.72	6.28
*PvTGA02*	Phvul.001G249300.1.v2.1	Chr01:50020192.50024885	1938	362	nucl:14	40.91	8.41
*PvTGA06*	Phvul.008G169000.1.v2.1	Chr08:47255536.47260171	1,486	369	nucl:14	41.75	6.38
*PvTGA04*	Phvul.007G003600.1.v2.1	Chr07:227318.234734	1824	444	nucl:14	49.11	6.18
*PvTGA01*	Phvul.001G123300.1.v2.1	Chr01:34014908.34021329	2025	460	nucl:14	50.82	8.53
*PvTGA05*	Phvul.007G025500.1.v2.1	Chr07:1911388.1914860	1805	462	nucl:14	51.46	6.03
*PvTGA03*	Phvul.003G028800.1.v2.1	Chr03:2892109.2896986	1,693	462	nucl:14	51.49	6.60
*PvTGA08*	Phvul.011G203400.1.v2.1	Chr11:51907497.51917127	1942	467	nucl:14	51.71	6.13

### 3.2 Conserved domain and gene structure of *PvTGA* genes

**FIGURE 2:**
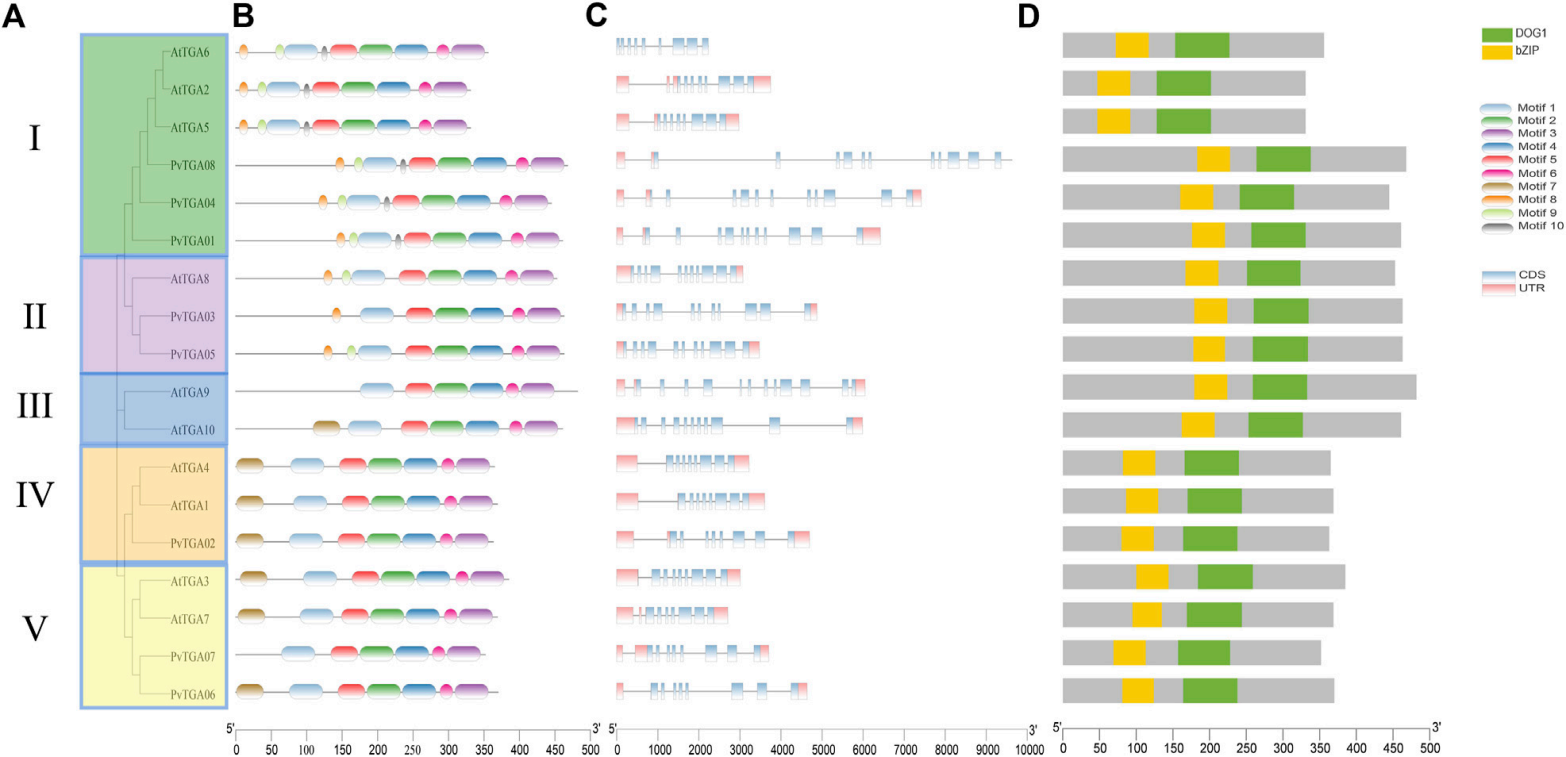
Phylogenetic, conserved motifs and gene structure of the *TGA* genes in *P. vulgaris* and *Arabidopsis*. **(A)** phylogenetic tree of the TGA protein sequence in common bean and *Arabidopsis* species constructed by the Maximum-Likelihood (ML) method. **(B)** conserved motifs in *PvTGAs* using MEME-suite. Various colors represented different motifs. **(C)** the eight common bean *TGA* gene structures. **(D)** comparison of conserved domain among *AtTGA* and *PvTGA*.The ruler at the bottom indicates length of the sequences.

To further analyze the evolutionary relationship between *TGA* genes in common bean, Maximum-Likelihood (ML) method was used to construct phylogenetic trees using common bean and *Arabidopsis* TGA protein sequence. As shown in Figure 2A, 8 *PvTGA* and 10 *AtTGA* divided into five subgroups, *PvTGAs* were distributed in four groups, *PvTGA01*, *PvTGA04*, and *PvTGA08* belonged to Clade I, *PvTGA03* and *PvTGA05* to Clade II, *PvTGA02* to Clade IV, *PvTGA06* and *PvTGA07* to Clade V, there was no common bean *TGA* gene in group III. Using the MEME online program, conserved motifs in the eight PvTGA proteins were found. Nine motifs, numbered from 1 to 10, totaling 11 to 50 amino acids in length, were found in the peanut proteins (Figure 2B; Supplementary Figure S1). The number of motifs contained in TGA protein ranged from six to nine and all of them contained six conserved motifs from Motif 1 to Motif 6. There are different motif distribution characteristics in different clade groups, such as motif 10 only exists in Clade II; Motif 8 and Motif 9 only exist in clade II and V, and Motif 7 is only identified in groups I, III and IV. The different distribution of motifs in different clades may lead to changes in the structure of the *TGA* genes, which in turn may determine the differentiation of different clade functions. To understand the connection between genomic evolution and functional differentiation, gene structure analysis was crucial. The number of exons in the *PvTGA* gene family is 9–15. From the evolutionary relationship, the *TGA* genes with close evolutionary relationship not only have the same number of exons, but also have similar structures (Figure 2C). A bZIP and DOG1 domain was found in every PvTGA protein, and these domains were found in the same relative locations across various sequences, according to a conserved structure study (Figure 2D). These results imply that the *PvTGA* gene has the same conserved domain in the gene structure, but there are specific sequence structures in different types of *TGA*.

### 3.3 Phylogenetic analysis of *PvTGA* genes

**FIGURE 3:**
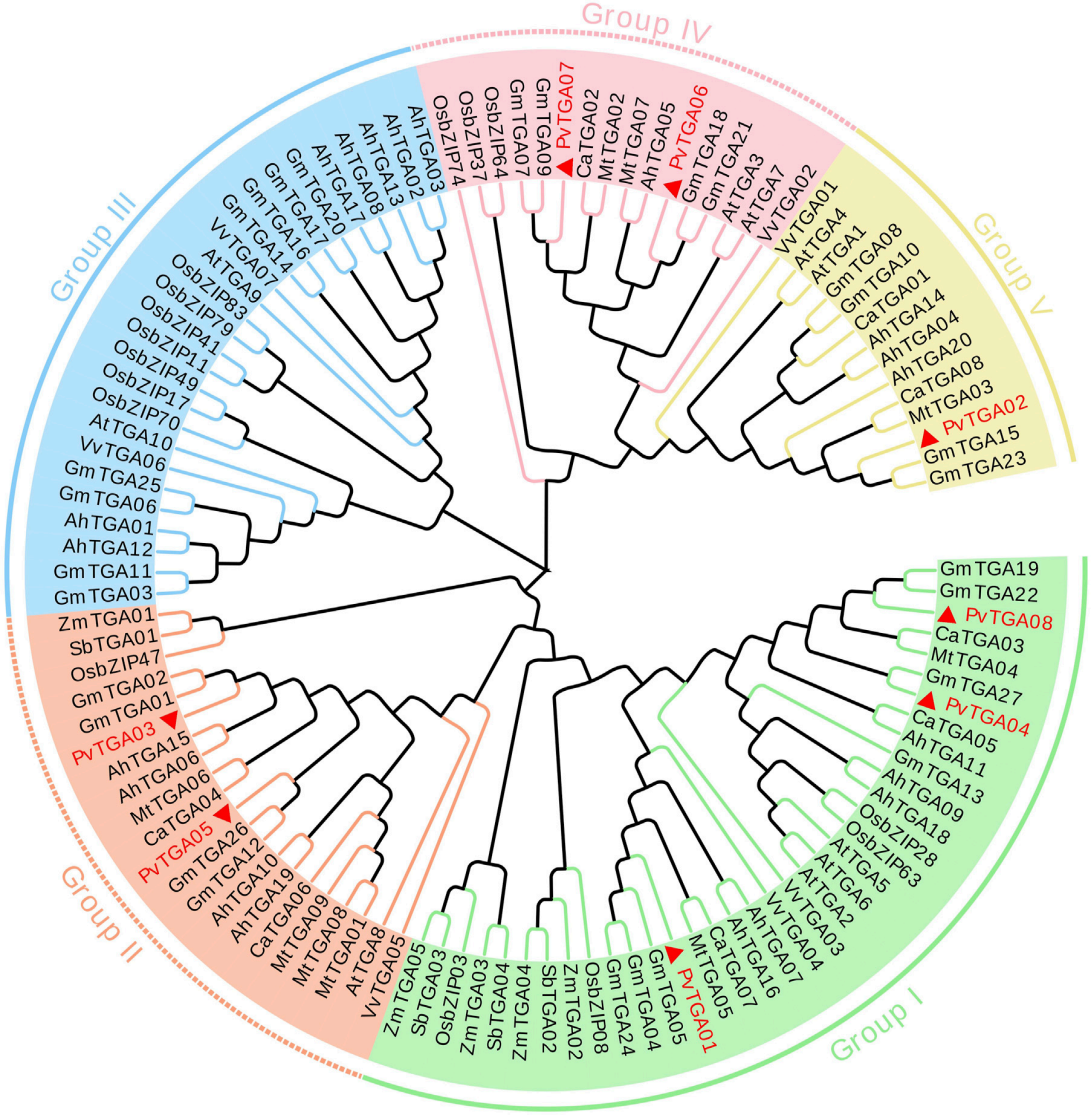
Evolutionary relationship analysis of TGA proteins from common bean (*Phaseolus vulgaris*; Pv). TGA amino acid sequences from the *Phaseolus vulgaris*, *Arabidopsis* (At), *Arachis hypogaea* (Ah), *Glycine max* (Gm), *Cicer arietinum* (Ca), rice (Os), corn (Zm), *Vitis vinifera* (Vv), *Medicago truncatula* (Mt) and sorghum (Sb) were used to construct a phylogenetic tree. Green line, orange line, blue line, pink line and yellow line represent group I, group II, group III, group IV and group V respectively.

Protein homology and cluster analysis were used to build phylogenetic trees of *PvTGA* genes from various species, and homology relations with other species were identified. In order to further study the evolutionary relationship of *TGA* family genes in different species, the MEGAX software was used to analyze the relationship between common bean, *Arabidopsis*, peanut (*Arachis hypogaea*), soybean (*Glycine max*), grape (*Vitis vinifera*), alfalfa (*Medicago sativa*), chickpea (*Cicer arietinum*), rice (*Oryza sativa*), maize (*Zea mays*) and Sorghum (*Sorghum bicolor*) TGA protein sequences constructed a phylogenetic tree (Supplementary Table S1). One hundred and thirteen TGA protein sequences were selected for multiple sequence alignment and a phylogenetic tree was constructed (Figure 3). *TGA*s were divided into five different subgroups, and the eight *PvTGAs* were assigned to four different groups, namely Group I, Group II, Group IV and V. Among these five subgroups, the number of *TGA* genes in the group I subgroup was 36. In the second subgroup, there are 18 genes in total, and in subgroup III, there are 26 members in total. In the subgroup IV, a total of 13 genes were included, and the subgroup V contained a total of 14 *TGAs*. The *PvTGAs* has a very close genetic distance with other legumes, especially soybean. The PvTGAs Proteins have the highest sequence similarity with those in soybean. Therefore, PvTGAs in common bean may have similar functions to the TGAs in soybean.

### 3.4 Synteny and duplication analysis of *PvTGA* genes

**FIGURE 4:**
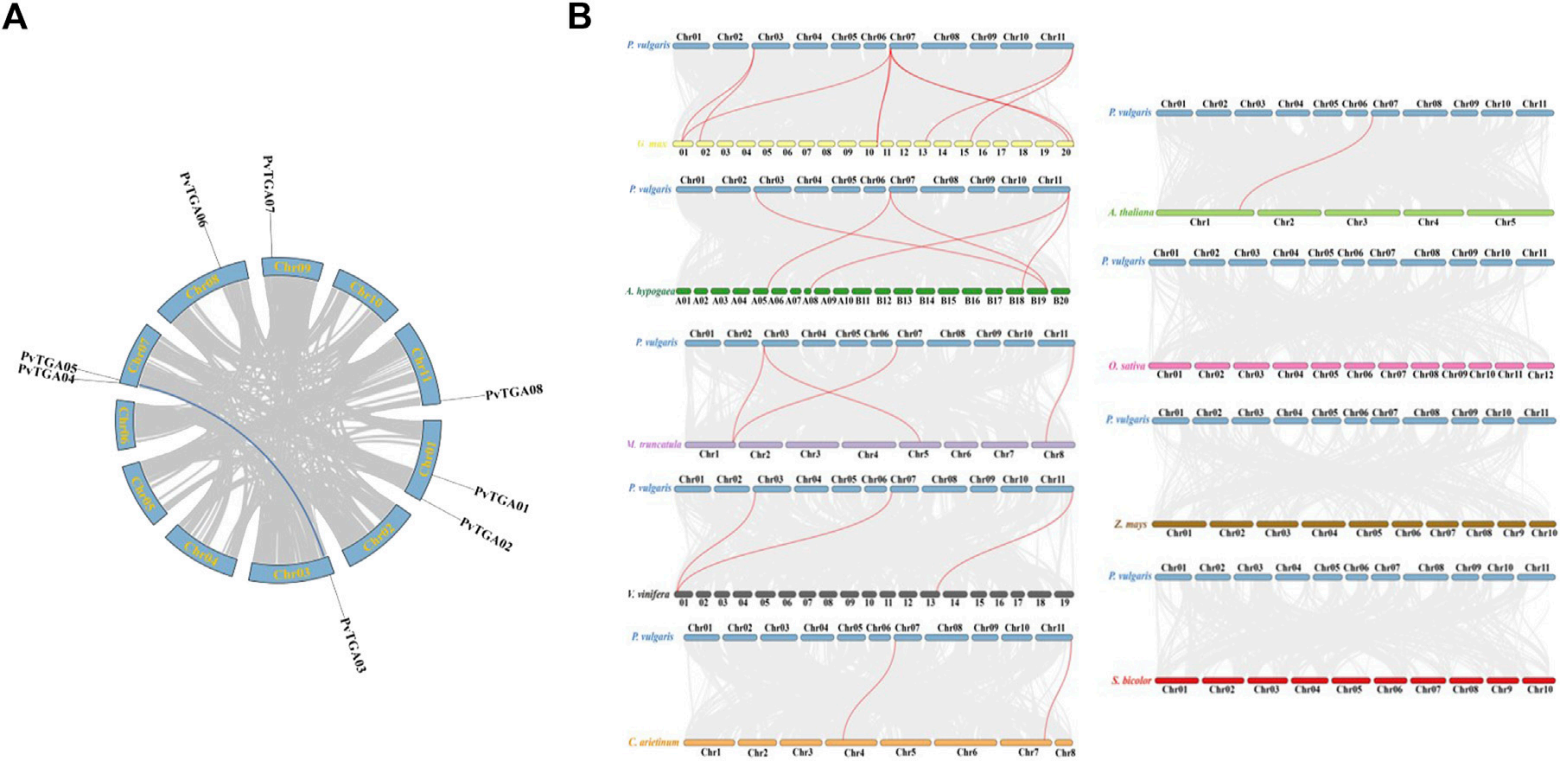
Genome scale synteny analysis of *Phaseolus vulgaris*, *Arabidopsis*, *Glycine max*, *Vitis vinifera*, *Medicago truncatula*, *Cicer arietinum*, rice, corn and sorghum *TGA* genes. **(A)** syntenic relationship of *P. vulgaris TGA* genes. All 8 *TGA* genes are marked according to their chromosome distribution in common bean genome and syntenic gene pairs are connected with blue lines., **(B)** syntenic pairs of *TGA* genes between *P. vulgaris* and other plants. The *Phaseolus vulgaris* (*P. vulgaris*), *Arabidopsis thaliana* (*A. thaliana*), *Arachis hypogaea* (*A. hypogaea*), *Glycine max* (*G. max*), *Cicer arietinum* (*C. arietinum*), *Vitis vinifera* (*V. vinifera*), *Medicago truncatula* (*M. truncatula*), *Oryzae Sativa* (*O. Sativa*), *Zea mays* (*Z. mays*) and *Sorghum bicolor* (*S. bicolor*) chromosomes are shown in blue, lightgreen, green, yellow, orange, grey, purple, pink, brown and red bars, respectively. Gray lines in the background indicated the collinear blocks within *P. vulgaris* and other plant genomes, while the Syntenic *TGA* gene pairs are linked with red lines.

Segmental duplication and tandem repeats were identified in common bean genome. The *TGA* gene in common bean does not have a tandem repeat event, but *PvTGA03* and *PvTGA05* are segmental duplications (Figure 4A). To further explore the evolutionary relationship of *TGA* genes between common bean and other species, the synteny relationship between *TGAs* and homologues of other species was investigated. Orthologous gene pairs were identified between *TGAs* of common bean and other plants, including soybean, chickpea, alfalfa, peanut, *Arabidopsis*, grape, rice, sorghum, and maize (Figure 4B). Common bean and other legumes including soybean, alfalfa, chickpea and peanut have gene pairs of 9, 4, 2, and 5, respectively; dicotyledons grape and *Arabidopsis* have gene three and one pairs with common bean, and monocots sorghum, rice and maize do not have any gene pairs with common bean. The results showed that *TGAs* in common bean were genetically closely related to ones in soybean, alfalfa, chickpea, and cultivated peanut, and had distant phylogenetic relationships with *Arabidopsis* and grapevine. There were no collinear gene pairs of *TGA*s between common bean and rice, sorghum and maize, suggesting a long-distance relationship between these species and common. *PvTGA03* and *PvTGA05* in the group II had the most homologous gene pairs with other species (Supplementary Table S2). These two genes co-exist in 6 to 9 gene pairs in common bean and other species, while other *TGA* genes only have 2 to 7 gene pairs. These results suggest that *PvTGA03* and *PvTGA05* may play key roles in the *TGA* gene family during evolution and have important functions.

To analyze the evolutionary selection pressure of *TGA* genes, we calculated the Ka/Ks ratio of *TGA* gene pairs. The Ka/Ks ratio of *TGA* collinear gene pairs between common bean and other species was less than 1, indicating that the *TGA* gene family was dominated by purifying selection during evolution (Supplementary Table S3).

### 3.5 *Cis*-element in the promoter region of the *PvTGA* genes

**FIGURE 5:**
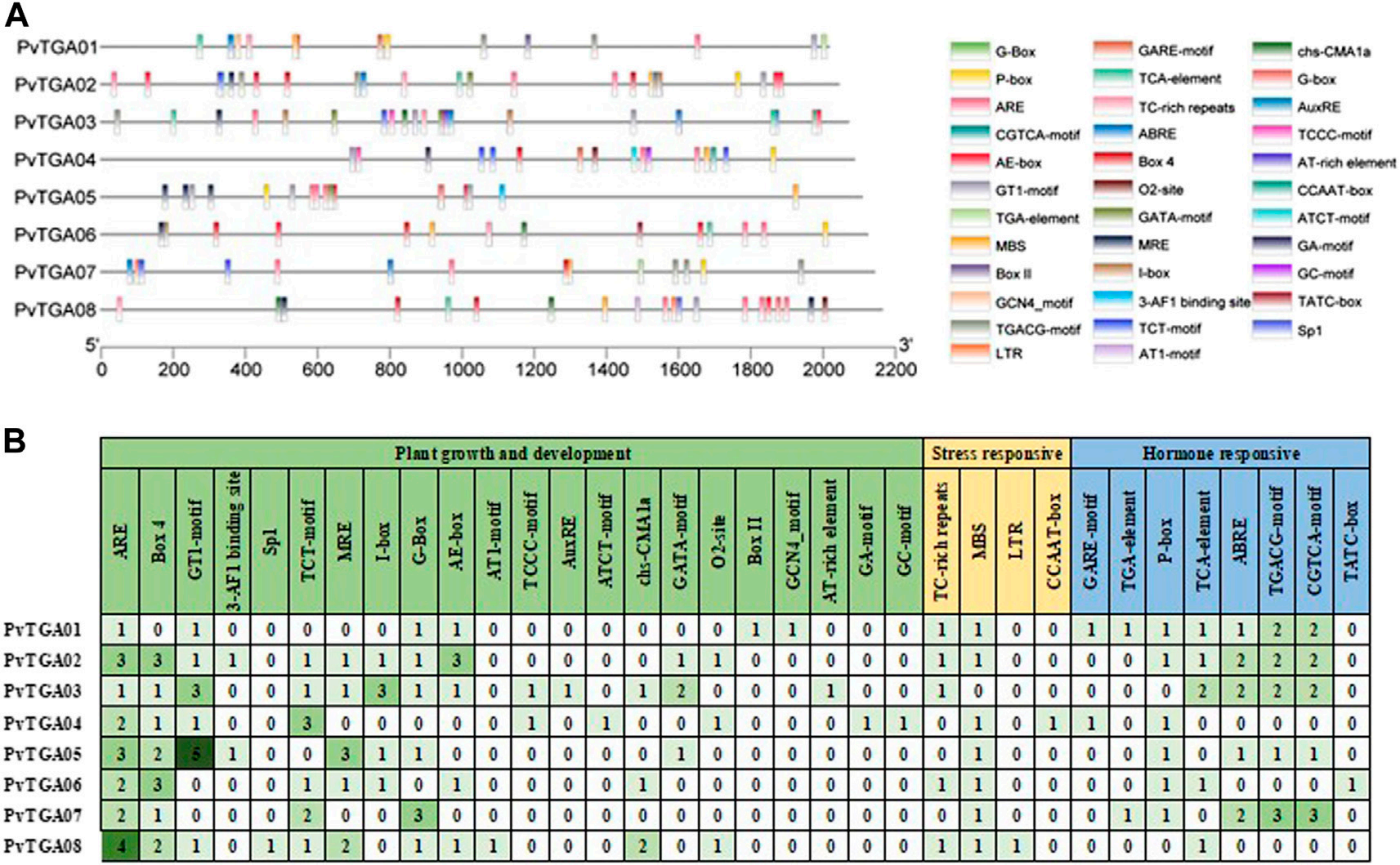
Analysis of the *cis*-acting elements in the promoter regions of 8 *TGA* genes. **(A)** the *cis*-acting elements distribution in 8 *TGA* genes promoters. Diverse colors were used for representing different *cis*-elements, as given in on the right side., **(B)** the names and numbers of cis-acting elements in 8 *TGA* genes promoters. The heatmap in grid and the color columns indicated the numbers of *cis*-acting elements.

The promoter activities are essential for controlling how genes operate. The *cis*-elements in the promoter regions of *TGA* genes are examined in order to comprehend the genetic processes, metabolic networks, and regulatory mechanisms involved (Figure 5). In total, 164 *cis*-acting elements of 34 types were predicted to contain potential functions (Supplementary Table S4). It was found 22 *cis* elements related to growth and development, eight hormone-related *cis*-elements and four stress-responsive-related elements. There are differences in the number and distribution of *cis*-elements in each gene (Figure 5A). The *PvTGA02* and *PvTGA03* promoters contain the most cis-acting elements, with 27, while the *PvTGA06* promoter contains the least *cis*-acting elements, only 15 (Figure 5B). Among the growth and development-related elements, the number of ARE elements on the promoter of *PvTGA* gene is the largest, followed by Box 4 and GT1-motif; Among the stress-related elements, the number of MBS was the largest, and the number of LTR and CCAAT-box elements was the least; among the hormone-related induction elements, the number of TGACG-motif and CGTCA-motif elements was the largest, and the number of TATC-box was the least. The analysis of *cis*-acting elements showed that *PvTGAs* may be involved in plant growth and development and responses to various hormones and stresses.

### 3.6 Expression profiling of *PvTGA* genes in different tissues

**FIGURE 6:**
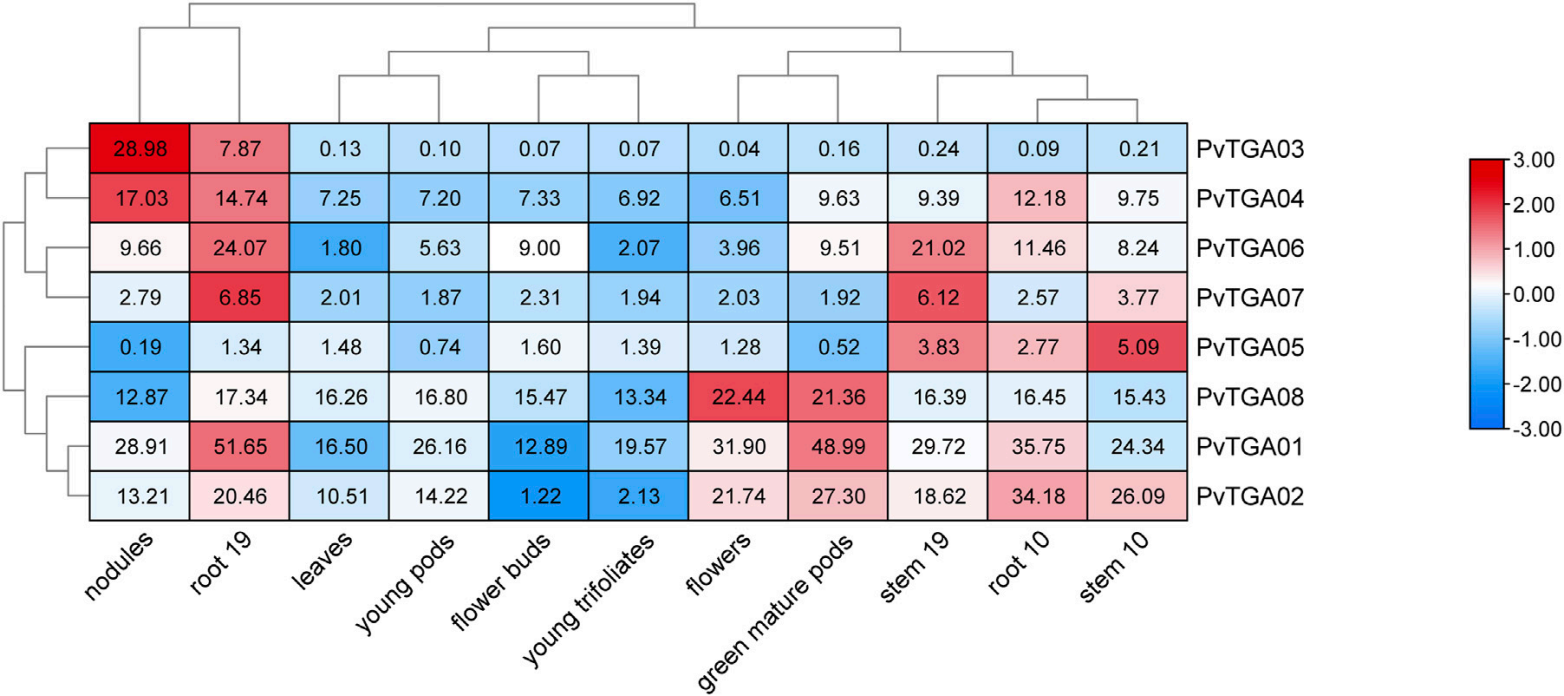
Expression profiles of *PvTGA* genes from common bean in 11 different tissues. Heat-map clustering based on FPKM expression of 8 *PvTGA* genes in different common bean tissues. Log2-transformed values are used in a color-coded heatmap, with bars representing gene-normalized FPKM (Log2) expression levels, and red for higher expression, blue means low.

In this study, we additionally aimed to perform mRNA analysis of *TGA* genes using publicly available expression data (https://phytozome.jgi.doe.gov). The heatmap, which was derived from mRNA levels, shows the expression variance of identified eight *PvTGA* genes in different plant tissues (Figure 6). All genes were expressed at low levels in flower buds, young pods, and young trifoliates, while most genes were expressed at high levels in roots. The expression levels of some genes were tissue-specific, for example, *PvTGA03* and *PvTGA04* were highly expressed in nodules and root 19, while *PvTGA1*, *PvTGA0*2 and *PvTGA08* were highly expressed in flowers and green muture pods. Varying tissues and developmental stages had different *PvTGA* expression levels, which suggested that these *TGA* genes were engaged in various developmental and regulatory processes in the common bean.

### 3.7 Phenotypic evaluation of common bean plants under *Fop* infection

**FIGURE 7:**
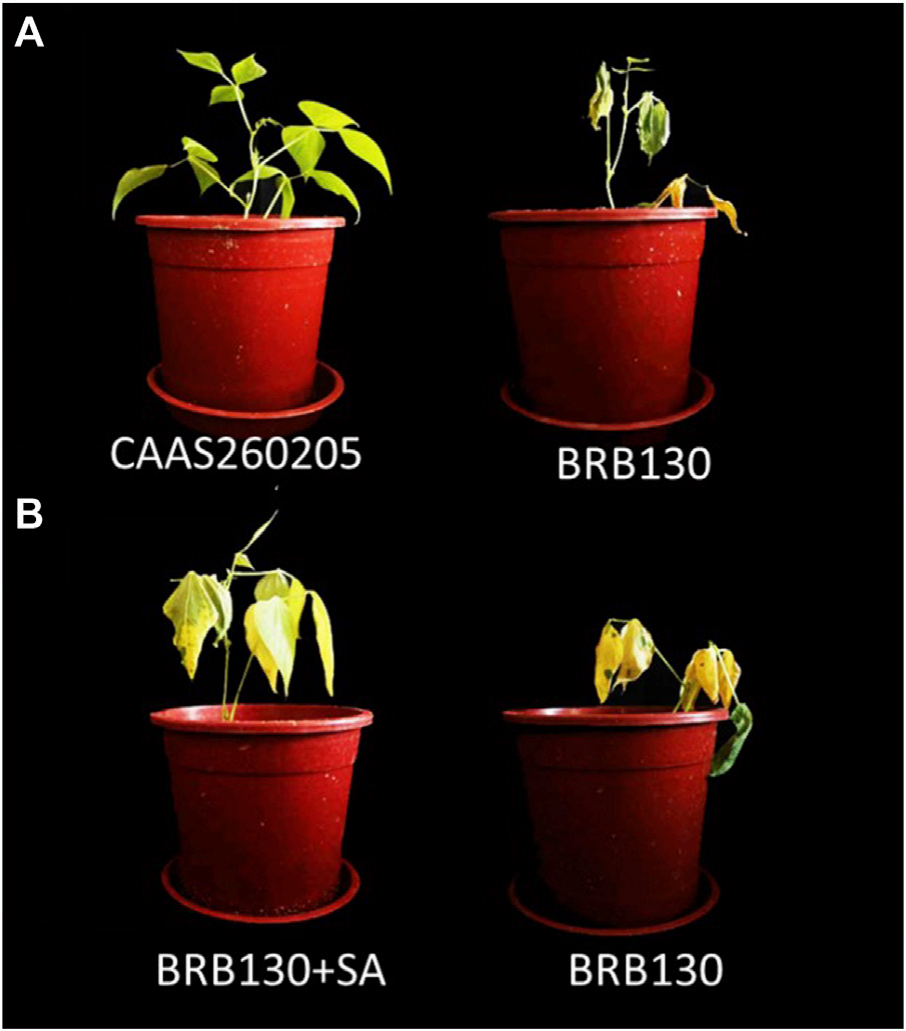
Phenotype of common bean genotype under *Fop* infection. **(A)** resistant genotype CAAS260205 and susceptible genotype BRB130 were tested with *Fop* isolate for 24 days., **(B)** susceptible genotype BRB130 (left) under five consecutive days of exogenous SA treatment and BRB130 without SA application (right) as control were inoculated with *Fop*.

The resistant genotype CAAS260205 and the susceptible genotype BRB130 were inoculated with *Fop* 24 days later, the susceptible genotype BRB130 showed vascular bundle necrosis and caused shoot wilting, while the resistant genotype CAAS260205 did not show vascular bundle necrosis and shoot wilting (Figure 7A). After five consecutive days of exogenous SA, the susceptible genotype BRB130 was inoculated with *Fop*. Twenty-four days after inoculation, exogenous SA can significantly alleviate the necrosis of vascular bundles in common bean plants and reduce the water loss in the shoot, while BRB130 plants without external application of SA obvious symptoms of wilting appear (Figure 7B).

### 3.8 Expression analysis of the *PvTGAs* under *Fop* infection

**FIGURE 8:**
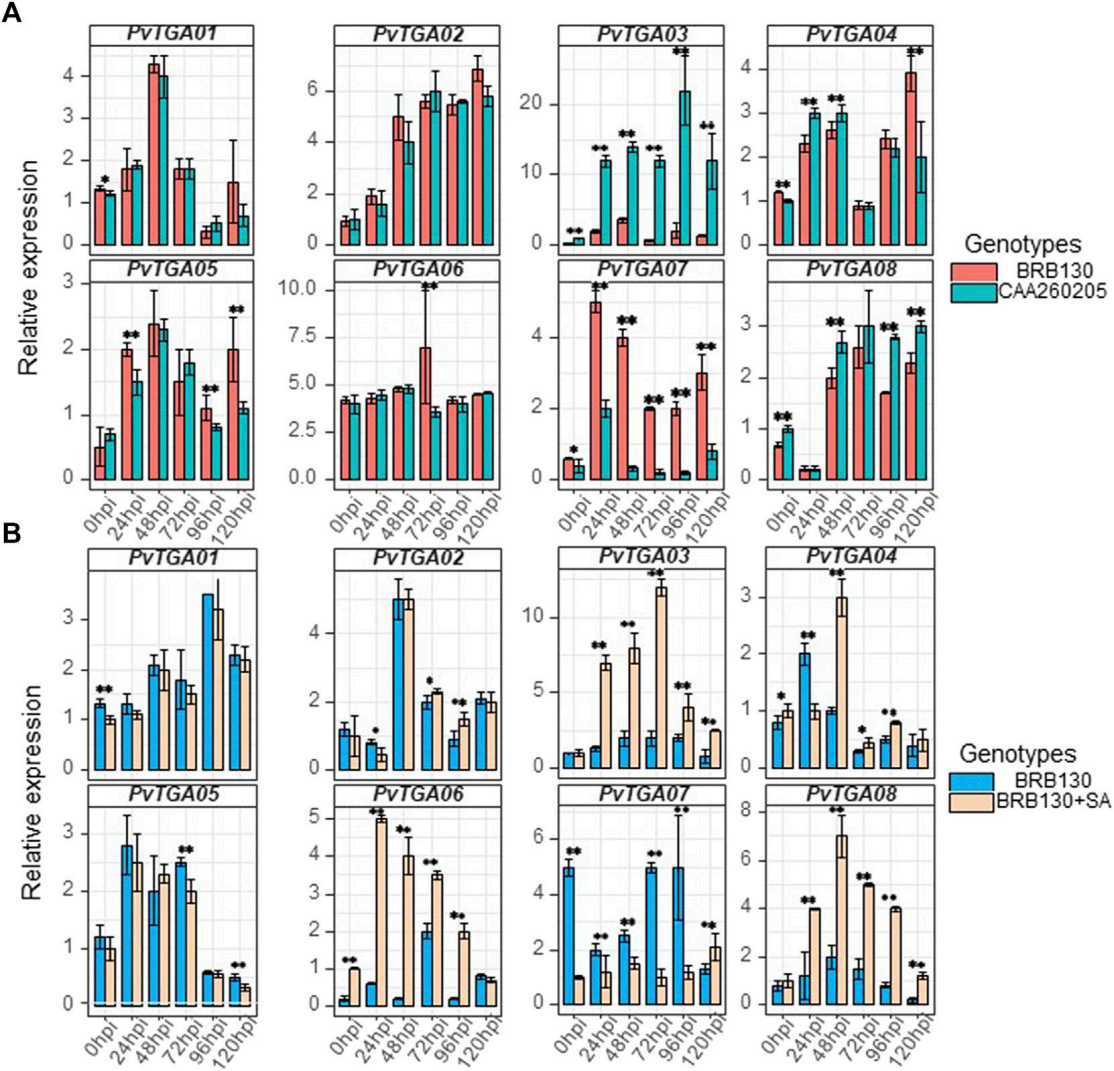
Differential expression of eight candidate genes in differernt varieties and processing. **(A)** relative expression of eight genes in susceptible (BRB130) and resistant (CAA260205) genotype at different time points after inoculation with *Fop isolates*., **(B)** relative expression of eight genes in susceptible genotype (BRB130) and sprayed with SA (BRB130 + SA) at different time points after inoculation with *Fop isolates*. (*, *p* < 0.05; **, *p* < 0.01; Student’s t-test).

Science Fusarium wilt is a typical soil-borne disease, the roots of common plants were collected to detect the expression level of *TGA* gene. Root tissues were selected for qRT-PCR examination and expression level analysis for eight *PvTGA* genes in the susceptible (BRB130) and resistant (CAAS260205) genotypes (Figure 8A). After inoculation with *Fop*, six time points were selected for expression level analysis using qPCR. Specific primers were designed for each *PvTGA* gene to detect gene expression levels (Supplementary Table S5). The expression levels of eight genes showed significant changes within 120 h after inoculation with *Fop* (Figure 8A). Between the resistant and susceptible genotypes, *PvTGA03* and *PvTGA07* showed significant differences in expression levels at all time points except 0 hpi between the resistant and susceptible genotypes. The expression level of *PvTGA03* in the resistant genotype CAAS260205 was significantly higher than that in the susceptible genotype BRB130, on the contrary, the expression level of *PvTGA07* in the resistant genotype was significantly lower than that in the susceptible genotype. This indicated that these two genes played opposite roles in the response to *Fop* resistance. Increased expression of *PvTGA03* promoted common bean disease resistance, while increased expression of *PvTGA07* promoted *Fop* infection and led to plant disease.

Previous studies have revealed that TGA protein plays a key role in the salicylic acid-mediated resistance pathway. To explore the molecular mechanism of *TGA* gene response to salicylic acid in common bean, the expression levels of eight *TGA* genes were examined under SA treatment using qRT-PCR. After SA treatment of common bean seedlings, some *TGA* genes did not show significant changes in expression levels, including *PvTGA01*, *PvTGA02* and *PvTGA05,* while *PvTGA03*, *PvTGA04*, *PvTGA06*, *PvTGA07* and *PvTGA08* were all showed significant changes in expression after SA treatment (Figure 8B). The disease resistance of common bean plants was enhanced after being treated with SA, and the expression levels of *PvTGA0*3, *PvTGA06* and *PvTGA08* were increased, while the expression level of *PvTGA07* was decreased. Therefore, these genes may be involved in SA-mediated disease resistance.

## 4 Discussion

The defensive response to invading pathogens is all physiological processes in plants that depend on the transcriptional control of gene expression. Through their entire life cycle, plants are controlled by TFs. TFs regulate a wide range of elements of plant growth and development, including flower and root development, and morphological diversity (Suter, 2020; Strader et al., 2022). Therefore, understanding the structure and function of TFs is crucial to comprehending how plant growth and development are regulated. The *TGA* genes were among the first plant TFs to be identified, and their structure and function have undergone significant research (Pontier et al., 2001; Gatz, 2013). However, related research on the *TGA* gene family found in common beans is still insufficient. In this study, *TGA* genes in common bean were identified genome-wide, and gene structure and genetic evolution relationships were explored. The expression pattern of the *PvTGA* gene was explored including differences in expression levels of different tissues, in resistant and susceptible genotypes, and after exogenous salicylic acid application. This work offers useful data for investigating these molecular processes and advancing common bean resistance breeding.

The TGA protein in common bean are similar to *Arabidopsis thaliana*, and can be divided into four clade, lacking *PvTGA* similar to *Arabidopsis thaliana* subgroup III *AtTGA9* and *AtTGA10.* Exon/intron and motif analysis revealed that the *PvTGA* members of various groups had considerably diverse gene architectures and lengths of sequences. The sensitivity of gene transcriptional regulation is often correlated with the number of introns. The capacity of the plant to respond to various developmental stages and environmental situations increases with decreasing intron number. The genes without introns or with shorter introns are also one of the active selection propensities of plants in evolution, however, in plants, animals and yeast, introns not only affect transcription to increase the content of mRNA, but also improve the translation efficiency of mRNA (Mattick and Gagen, 2001; Shaul, 2017). *PvTGA* genes have different annotated intron counts. *PvTGAs* have a certain amount of introns as a result of long-term evolution. *PvTGAs* may have different biological activities as a result of their various intron architectures. After conserved domain and motif analysis, it was revealed that all the *PvTGAs* shared the typical motifs, and motif sequences of each subfamily were relatively similar, indicating that members in each *TGA* subfamily might have the same function (Kesarwani et al., 2007). All TGA proteins contain key motifs that consist of the bZIP and DOG1 domains, but contain unique motifs in different subgroups. Genes’ functions are determined by their physical and chemical characteristics. PvTGA proteins’ physicochemical characteristics demonstrated their abundance in acidic amino acids (Table 1). The bZIP domain of PvTGA proteins was generally conserved, as shown by protein structure predictions and sequence alignment. Additionally, research has demonstrated that the specificity of DNA and nuclear localization is determined by the alkaline region of the bZIP domain’s interaction with DNA *via* the fixed nuclear localization signal structure NX7-R/K-X9 (Dröge-Laser et al., 2018). In accordance with *TGA* transcription factors described in other plants (Idrovo Espín et al., 2012; Xu et al., 2018; Li et al., 2019; Liu et al., 2022), subcellular localization revealed that the *PvTGA* transcription factors in common bean were mostly found in the cell nucleus, demonstrating a critical function for the *PvTGA* transcription factors in the nucleus. It was suggested that while the functions of the genes in various branches varied, the functions of the gene family were conserved.

The evolutionary process of orthologous and paralogous genes has an important relationship to the function of gene families (Koonin, 2005; Gabaldón and Koonin, 2013). The 512 Mb genome of common bean contains eight *TGA* genes, and the identification results based on segmental duplication and tandem repeats show that only *PvTGA03* and *PvTGA05* are caused by segmental duplication, and no tandem repeats occur among *PvTGAs*, which may be the *TGA* gene in the common genome is relatively conservative. The values of all *TGA* genes in Ka/Ks analysis were less than 1, which also indicated that the *PvTGA* genes was dominated by purifying selection. Different plant species have different numbers of *TGA* genes (Idrovo Espín et al., 2012; Xu et al., 2018; Li et al., 2019; Liu et al., 2022). The genome sizes of soybean, chickpea, alfalfa, peanut, *Arabidopsis*, grape, rice, sorghum, and maize are 967, 525, 408 Mb, 2.5 Gb, 118, 482, 373, 704 Mb, 2.1 Gb, respectively (https://phytozome-next.jgi.doe.gov/). The number of *TGA* genes identified in different plants is 10 in *Arabidopsis*, 20 in peanut, 9 in alfalfa, 27 in soybean, 4 in sorghum, 15 in rice, 5 in maize, eight in chickpea, and 7 in grape (Li et al., 2019; Liu et al., 2022). Thus, the genome sizes in these plants are not correlated with the number of *TGA* genes. Synteny analysis showed that *PvTGAs* had a closer genetic relationship with legumes, but a farther relationship with monocots, among which the *TGA* gene in soybean was the closest to the ones in common bean, and had the most orthologous gene pairs. It shows that the *TGA* genes in common bean and soybean may have similar functions. These results are consistent with the phylogenetic relationship between common bean and the other species. *PvTGA03* and *PvTGA05* produced by segmental duplication have the most homologous gene pairs in other species, indicating that these two genes may play an important role in the evolution of *TGA* genes (Fulton et al., 2002).

In this study, CAAS260205, a resistant genotype that highly expresses SA to provide effective SAR against *Fop*, was used together with the susceptible genotype BRB130 to detect *TGA* gene responses after inoculation. The results showed that *PvTGA03* and *PvTGA07* play opposite roles to regulate SA-mediated resistance to *Fusarium wilt*. And these two genes were highly expressed in the root of common bean, which can provide better sensitivity for the response to *Fusarium wilt* infection. Interestingly, the soybean *TGA* genes closest to these two genes in phylogenetic relationship were not found to be stress-responsive to biotic and abiotic stress (Li et al., 2019; Ullah et al., 2019; Jiang et al., 2021). *AtTGA8* can control flowering-related morphological traits (Chuang et al., 1999), while *PvTGA03* is associated with SA-mediated disease resistance. *AtTGA3* interacts with ARR2 and binds to the PR1 promoter under the action of cytokinin (CTK) to improve plant disease resistance, but *PvTGA07* plays a negative regulatory role in the process of resistance to Fusarium wilt (Choi et al., 2004; Fang et al., 2017). The role of the same *TGA* subgroup among different species might be highly diverse (Zhang et al., 2003; Fitzgerald et al., 2005; Thurow et al., 2005). While silencing *TGA2.1* in tobacco led to petal-like stamens of *TGA2.1*, which is important for increasing the resistance to pathogens in silenced rice, knocking out *TGA2* in *Arabidopsis thaliana* resulted in no phenotype of *TGA2*. Therefore, the anti-*Fop* response mechanism of *TGA* mediated by SA may be different from that of other plant *TGA* genes, and further in-depth functional studies on *PvTGA* genes are needed to analyze the molecular mechanism.

In summary, the findings of this study lay the groundwork for future investigation into the function of the *TGA* gene family in the regulation of common bean growth, development, and disease resistance. They also serve as a guide for the potential use of the *PvTGA* gene family in common bean resistance breeding.

## Data Availability

The datasets presented in this study can be found in online repositories. The names of the repository/repositories and accession number(s) can be found in the article/Supplementary Material.

## References

[B1] An C. Mou Z. (2011). Salicylic acid and its function in plant immunity. J. Integr. Plant Biol. 53 (6), 412–428. 10.1111/j.1744-7909.2011.01043.x 21535470

[B3] Bailey T. L. Williams N. Misleh C. Li W. W. (2006). Meme: Discovering and analyzing DNA and protein sequence motifs. Nucleic Acids Res. 34 (2), W369–W373. 10.1093/nar/gkl198 16845028 PMC1538909

[B4] Baillo E. H. Kimotho R. N. Zhang Z. Xu P. (2019). Transcription factors associated with abiotic and biotic stress tolerance and their potential for crops improvement. Genes. 10 (10), 771. 10.3390/genes10100771 31575043 PMC6827364

[B5] Bertioli D. J. Jenkins J. Clevenger J. Dudchenko O. Gao D. Seijo G. (2019). The genome sequence of segmental allotetraploid peanut *Arachis hypogaea* . Nat. Genet. 51, 877–884. 10.1038/s41588-019-0405-z 31043755

[B6] Bigeard J. Colcombet J. Hirt H. (2015). Signaling mechanisms in pattern-triggered immunity (PTI). Mol. Plant 8 (4), 521–539. 10.1016/j.molp.2014.12.022 25744358

[B7] Blair M. Izquierdo P. Astudillo C. Grusak M. (2013). A legume biofortification quandary: Variability and genetic control of seed coat micronutrient accumulation in common beans. Front. Plant Sci. 4, 275. 10.3389/fpls.2013.00275 23908660 PMC3725406

[B8] Blair M. W. Wu J. Wang S. (2016). Editorial: Food legume diversity and legume research policies. Crop J. 4 (5), 339–343. 10.1016/j.cj.2016.09.001

[B9] Buruchara R. A. Camacho L. (2000). Common bean reaction to Fusarium oxysporum f. sp. phaseoli, the cause of severe vascular wilt in central Africa. J. Phytopathology 148 (1), 39–45. 10.1111/j.1439-0434.2000.tb04622.x

[B10] Chen X. Lu Q. Liu H. Zhang J. Hong Y. Lan H. (2019). Sequencing of cultivated peanut, *Arachis hypogaea*, yields insights into genome evolution and oil improvement. Mol. Plant 12 (7), 920–934. 10.1016/j.molp.2019.03.005 30902685

[B11] Cheng C.-Y. Krishnakumar V. Chan A. P. Thibaud-Nissen F. Schobel S. Town C. D. (2017). Araport11: A complete reannotation of the *Arabidopsis thaliana* reference genome. Plant J. 89, 789–804. 10.1111/tpj.13415 27862469

[B12] Choi J. J. Klosterman S. J. Hadwiger L. A. (2004). A promoter from pea gene DRR206 is suitable to regulate an elicitor-coding gene and develop disease resistance. Phytopathology® 94 (6), 651–660. 10.1094/phyto.2004.94.6.651 18943490

[B13] Chuang C. F. Running M. P. Williams R. W. Meyerowitz E. M. (1999). The PERIANTHIA gene encodes a bZIP protein involved in the determination of floral organ number in *Arabidopsis thaliana* . Genes. Dev. 13 (3), 334–344. 10.1101/gad.13.3.334 9990857 PMC316427

[B14] Dempsey D. M. A. Klessig D. F. (2012). SOS – too many signals for systemic acquired resistance? Trends Plant Sci. 17 (9), 538–545. 10.1016/j.tplants.2012.05.011 22749315

[B15] Dröge-Laser W. Snoek B. L. Snel B. Weiste C. (2018). The Arabidopsis bZIP transcription factor family—An update. Curr. Opin. Plant Biol. 45, 36–49. 10.1016/j.pbi.2018.05.001 29860175

[B16] Fang H. Liu Z. Long Y. Liang Y. Jin Z. Zhang L. (2017). The Ca2+/calmodulin2-binding transcription factor TGA3 elevates LCD expression and H2S production to bolster Cr6+ tolerance in Arabidopsis. Plant J. 91 (6), 1038–1050. 10.1111/tpj.13627 28670772

[B17] Fitzgerald H. A. Canlas P. E. Chern M.-S. Ronald P. C. (2005). Alteration of TGA factor activity in rice results in enhanced tolerance to Xanthomonas oryzae pv. oryzae. Plant J. 43 (3), 335–347. 10.1111/j.1365-313X.2005.02457.x 16045470

[B18] Fu Z. Dong X. (2013). Systemic acquired resistance: Turning local infection into global defense. Annu. Rev. plant Biol. 64, 839–863. 10.1146/annurev-arplant-042811-105606 23373699

[B19] Fulton T. M. Van der Hoeven R. Eannetta N. T. Tanksley S. D. (2002). Identification, analysis, and utilization of conserved ortholog set markers for comparative genomics in higher plants. Plant Cell. 14 (7), 1457–1467. 10.1105/tpc.010479 12119367 PMC150699

[B20] Gabaldón T. Koonin E. V. (2013). Functional and evolutionary implications of gene orthology. Nat. Rev. Genet. 14 (5), 360–366. 10.1038/nrg3456 23552219 PMC5877793

[B21] Gatz C. (2013). From pioneers to team players: TGA transcription factors provide a molecular link between different stress pathways. Mol. Plant-Microbe Interactions® 26 (2), 151–159. 10.1094/mpmi-04-12-0078-ia 23013435

[B22] Idrovo Espín F. M. Peraza-Echeverria S. Fuentes G. Santamaría J. M. (2012). *In silico* cloning and characterization of the TGA (TGACG MOTIF-BINDING FACTOR) transcription factors subfamily in Carica papaya. Plant Physiology Biochem. 54, 113–122. 10.1016/j.plaphy.2012.02.011 22410205

[B23] Jakoby M. Weisshaar B. Dröge-Laser W. Vicente-Carbajosa J. Tiedemann J. Kroj T. (2002). bZIP transcription factors in Arabidopsis. Trends Plant Sci. 7 (3), 106–111. 10.1016/S1360-1385(01)02223-3 11906833

[B24] Jiang H. Gu S. Li K. Gai J. (2021). Two TGA transcription factor members from hyper-susceptible soybean exhibiting significant basal resistance to soybean mosaic virus. Int. J. Mol. Sci. [Online] 22 (21), 11329. 10.3390/ijms222111329 34768757 PMC8583413

[B25] Johnson C. Boden E. Desai M. Pascuzzi P. Arias J. (2001). *In vivo* target promoter-binding activities of a xenobiotic stress-activated TGA factor. Plant J. 28 (2), 237–243. 10.1046/j.1365-313X.2001.01147.x 11722767

[B26] Jones J. D. G. Dangl J. L. (2006). The plant immune system. Nature 444 (7117), 323–329. 10.1038/nature05286 17108957

[B27] Katagiri F. Lam E. Chua N.-H. (1989). Two tobacco DNA-binding proteins with homology to the nuclear factor CREB. Nature 340 (6236), 727–730. 10.1038/340727a0 2528073

[B28] Kesarwani M. Yoo J. Dong X. (2007). Genetic interactions of TGA transcription factors in the regulation of pathogenesis-related genes and disease resistance in Arabidopsis. Plant Physiol. 144 (1), 336–346. 10.1104/pp.106.095299 17369431 PMC1913812

[B29] Klessig D. F. Choi H. W. Dempsey D. M. A. (2018). Systemic acquired resistance and salicylic acid: Past, present, and future. Mol. Plant-Microbe Interactions® 31 (9), 871–888. 10.1094/mpmi-03-18-0067-cr 29781762

[B30] Koonin E. V. (2005). Orthologs, paralogs, and evolutionary genomics. Annu. Rev. Genet. 39 (1), 309–338. 10.1146/annurev.genet.39.073003.114725 16285863

[B31] Kumar S. Stecher G. Li M. Knyaz C. Tamura K. (2018). Mega X: Molecular evolutionary genetics analysis across computing platforms. Mol. Biol. Evol. 35 (6), 1547–1549. 10.1093/molbev/msy096 29722887 PMC5967553

[B32] Li B. Liu Y. Cui X.-Y. Fu J.-D. Zhou Y.-B. Zheng W.-J. (2019). Genome-wide characterization and expression analysis of soybean TGA transcription factors identified a novel TGA gene involved in drought and salt tolerance. Front. Plant Sci. 10, 549. 10.3389/fpls.2019.00549 31156656 PMC6531876

[B33] Liu W. Zhao C. Liu L. Huang D. Ma C. Li R. (2022). Genome-wide identification of the TGA gene family in kiwifruit (Actinidia chinensis spp.) and revealing its roles in response to *Pseudomonas syringae* pv. actinidiae (Psa) infection. Int. J. Biol. Macromol. 222, 101–113. 10.1016/j.ijbiomac.2022.09.154 36150565

[B34] Livak K. J. Schmittgen T. D. (2001). Analysis of relative gene expression data using real-time quantitative PCR and the 2(-Delta Delta C(T)) Method. Methods 25 (4), 402–408. 10.1006/meth.2001.1262 11846609

[B35] Malamy J. Carr J. P. Klessig D. F. Raskin I. (1990). Salicylic acid: A likely endogenous signal in the resistance response of tobacco to viral infection. Science 250(4983), 1002–1004. 10.1126/science.250.4983.1002 17746925

[B36] Mattick J. S. Gagen M. J. (2001). The evolution of controlled multitasked gene networks: The role of introns and other noncoding RNAs in the development of complex organisms. Mol. Biol. Evol. 18 (9), 1611–1630. 10.1093/oxfordjournals.molbev.a003951 11504843

[B37] McCormick R. F. Truong S. K. Sreedasyam A. Jenkins J. Shu S. Sims D. (2018). The sorghum bicolor reference genome: Improved assembly, gene annotations, a transcriptome atlas, and signatures of genome organization. Plant J. 93, 338–354. 10.1111/tpj.13781 29161754

[B38] Pérez-Vega E. Pañeda A. Rodríguez-Suárez C. Campa A. Giraldez R. Ferreira J. J. (2010). Mapping of QTLs for morpho-agronomic and seed quality traits in a RIL population of common bean (Phaseolus vulgaris L.). Theor. Appl. Genet. 120 (7), 1367–1380. 10.1007/s00122-010-1261-5 20084493

[B39] Pontier D. Miao Z.-H. Lam E. (2001). Trans-dominant suppression of plant TGA factors reveals their negative and positive roles in plant defense responses. Plant J. 27 (6), 529–538. 10.1046/j.1365-313X.2001.01086.x 11576436

[B40] Rochon A. Boyle P. Wignes T. Fobert P. R. Després C. (2006). The coactivator function of Arabidopsis NPR1 requires the core of its BTB/POZ domain and the oxidation of C-terminal cysteines. Plant Cell. 18 (12), 3670–3685. 10.1105/tpc.106.046953 17172357 PMC1785396

[B41] Schmutz J. McClean P. E. Mamidi S. Wu G. A. Cannon S. B. Grimwood J. (2014). A reference genome for common bean and genome-wide analysis of dual domestications. Nat. Genet. 46 (7), 707–713. 10.1038/ng.3008 24908249 PMC7048698

[B42] Schwartz H. F. Steadman J. R. Hall R. (2005). Compendium of bean diseases. 2nd ed. St. Paul, MN: The American phytopathological Society.

[B43] Shaul O. (2017). How introns enhance gene expression. Int. J. Biochem. Cell. Biol. 91, 145–155. 10.1016/j.biocel.2017.06.016 28673892

[B44] Shimizu K. Suzuki H. Uemura T. Nozawa A. Desaki Y. Hoshino R. (2022). Immune gene activation by NPR and TGA transcriptional regulators in the model monocot Brachypodium distachyon. Plant J. 110 (2), 470–481. 10.1111/tpj.15681 35061931

[B45] Spoel S. H. Dong X. (2012). How do plants achieve immunity? Defence without specialized immune cells. Nat. Rev. Immunol. 12 (2), 89–100. 10.1038/nri3141 22273771

[B46] Strader L. Weijers D. Wagner D. (2022). Plant transcription factors — Being in the right place with the right company. Curr. Opin. Plant Biol. 65, 102136. 10.1016/j.pbi.2021.102136 34856504 PMC8844091

[B47] Suárez-Martínez S. E. Ferriz-Martínez R. A. Campos-Vega R. Elton-Puente J. E. de la Torre Carbot K. García-Gasca T. (2016). Bean seeds: Leading nutraceutical source for human health. CyTA - J. Food 14 (1), 131–137. 10.1080/19476337.2015.1063548

[B48] Suter D. M. (2020). Transcription factors and DNA play hide and seek. Trends Cell. Biol. 30 (6), 491–500. 10.1016/j.tcb.2020.03.003 32413318

[B49] Tang H. Krishnakumar V. Bidwell S. Rosen B. Chan A. Zhou S. (2014). An improved genome release (version Mt4.0) for the model legume Medicago truncatula. BMC Genomics 15, 312. 10.1186/1471-2164-15-312 24767513 PMC4234490

[B2] The French‐Italian Public Consortium for Grapevine Genome Characterization (2007). The grapevine genome sequence suggests ancestral hexaploidization in major angiosperm phyla. Nature 449, 463–467. 10.1038/nature06148 17721507

[B50] Thurow C. Schiermeyer A. Krawczyk S. Butterbrodt T. Nickolov K. Gatz C. (2005). Tobacco bZIP transcription factor TGA2.2 and related factor TGA2.1 have distinct roles in plant defense responses and plant development. Plant J. 44 (1), 100–113. 10.1111/j.1365-313X.2005.02513.x 16167899

[B51] Ullah I. Magdy M. Wang L. Liu M. Li X. (2019). Genome-wide identification and evolutionary analysis of TGA transcription factors in soybean. Sci. Rep. 9 (1), 11186. 10.1038/s41598-019-47316-z 31371739 PMC6672012

[B52] Varshney R. Song C. Saxena R. Azam S. Yu S. Sharpe A. G. (2013). Draft genome sequence of chickpea (Cicer arietinum) provides a resource for trait improvement. Nat. Biotechnol. 31, 240–246. 10.1038/nbt.2491 23354103

[B53] Wang D. Zhang Y. Zhang Z. Zhu J. Yu J. (2010). KaKs_Calculator 2.0: A toolkit incorporating gamma-series methods and sliding window strategies. Genomics, Proteomics Bioinforma. 8 (1), 77–80. 10.1016/S1672-0229(10)60008-3 PMC5054116 20451164

[B54] Wang Y. Tang H. DeBarry J. D. Tan X. Li J. Wang X. (2012). MCScanX: A toolkit for detection and evolutionary analysis of gene synteny and collinearity. Nucleic Acids Res. 40 (7), e49. 10.1093/nar/gkr1293 22217600 PMC3326336

[B55] Xu Z. Zhang H. Mo Q. Lü S. Wang C. Ji W. (2018). Expression analysis of wheat transcription factor TaTGA1 gene responding to infection of powdery mildew. Acta Phytopathol. Sin. 48 (6), 766. 10.13926/j.cnki.apps.000189

[B56] Xue R. Feng M. Chen J. Ge W. Blair M. W. (2021). A methyl esterase 1 (PvMES1) promotes the salicylic acid pathway and enhances Fusarium wilt resistance in common beans. Theor. Appl. Genet. 134 (8), 2379–2398. 10.1007/s00122-021-03830-1 34128089

[B57] Xue R. F. Wu J. Wang L. F. Blair M. W. Wang X. M. De Ge W. (2014). Salicylic acid enhances resistance to Fusarium oxysporum f. sp. phaseoli in common beans (Phaseolus vulgaris L.). J. Plant Growth Regul. 33 (2), 470–476. 10.1007/s00344-013-9376-y

[B58] Xue R. Wu X. Wang Y. Zhuang Y. Chen J. Wu J. (2017). Hairy root transgene expression analysis of a secretory peroxidase (PvPOX1) from common bean infected by Fusarium wilt. Plant Sci. 260, 1–7. 10.1016/j.plantsci.2017.03.011 28554466

[B59] Xue R. Zhu Z. Huang Y. Wang X. Wang L. Wang S. (2012). Quantification of <I&gt;Fusarium oxysporum&lt;/I&gt; f. sp. <I&gt;phaseoli&lt;/I&gt; Detected by Real-time Quantitative PCR in Different Common Beans Cultivars. Acta Agron. Sin. 38 (5), 791–799. 10.3724/sp.j.1006.2012.00791

[B60] Zhang Y. Tessaro M. J. Lassner M. Li X. (2003). Knockout analysis of Arabidopsis transcription factors TGA2, TGA5, and TGA6 reveals their redundant and essential roles in systemic acquired resistance. Plant Cell. 15 (11), 2647–2653. 10.1105/tpc.014894 14576289 PMC280568

[B61] Zhou J.-M. Zhang Y. (2020). Plant immunity: Danger perception and signaling. Cell. 181 (5), 978–989. 10.1016/j.cell.2020.04.028 32442407

