# Supplementary material for: Genome-wide identification of the TGA genes in common bean (Phaseolus vulgaris) and revealing their functions in response to Fusarium oxysporum f. sp. phaseoli infection
Source: Front Genet. 2023 Jan 20;14:1137634. doi: 10.3389/fgene.2023.1137634 (PMC9901207; doi:10.3389/fgene.2023.1137634)
Supplement: Supplementary file 1 [file DataSheet1.docx]

**
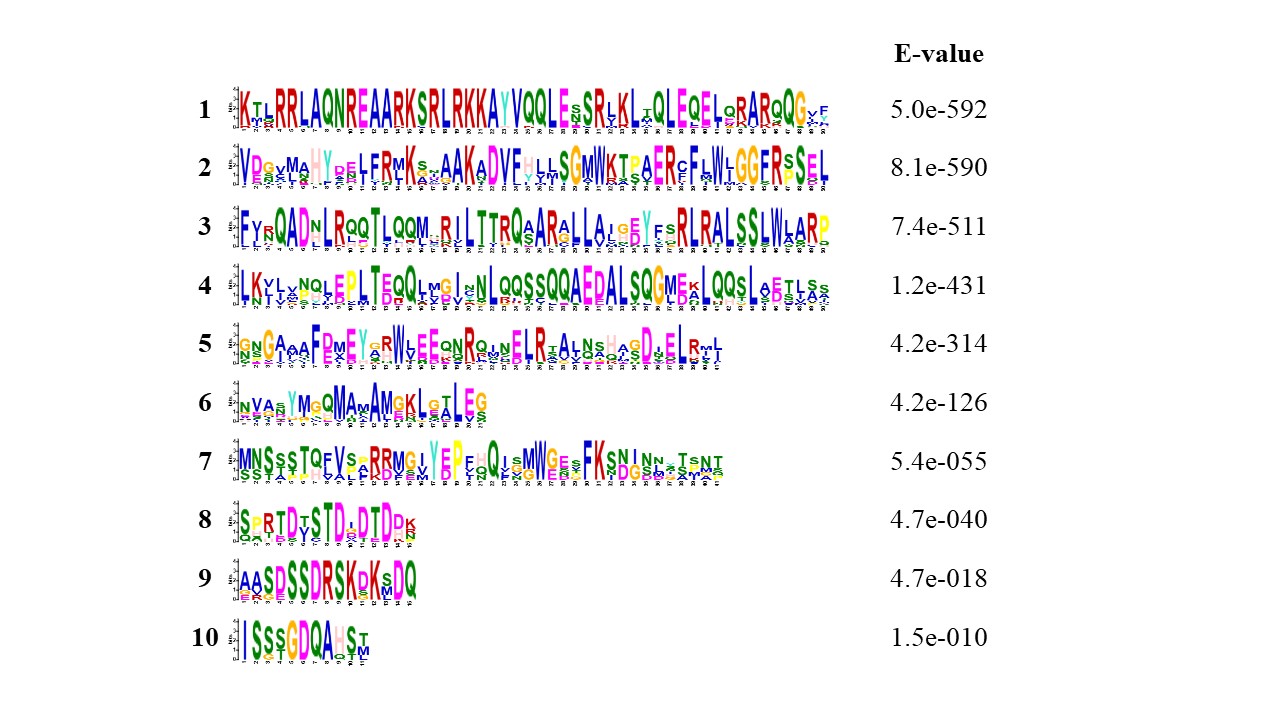
**

**Figure S1** Sequence logos of 10 motifs in common bean TGA proteins.

**Table S1:** List of the *TGA* genes ID identified in this study.

| **Species** | **Identifier** | **Accession number** | **TAIR-Locus ID** |
| --- | --- | --- | --- |
| *Arabidopsis thaliana* | AtTGA1 |  | AT5G65210.1.Araport11.447 |
|  | AtTGA2 |  | AT5G06950.1.Araport11.447 |
|  | AtTGA3 |  | AT1G22070.1.Araport11.447 |
|  | AtTGA4 |  | AT5G10030.1.Araport11.447 |
|  | AtTGA5 |  | AT5G06960.1.Araport11.447 |
|  | AtTGA6 |  | AT3G12250.4.Araport11.447 |
|  | AtTGA7 |  | AT1G77920.1.Araport11.447 |
|  | AtTGA8 |  | AT1G68640.1.Araport11.447 |
|  | AtTGA9 |  | AT1G08320.1.Araport11.447 |
|  | AtTGA10 |  | AT5G06839.3.Araport11.447 |
| *Phaseolus vulgaris* | PvTGA01 | Phvul.001G123300.1.v2.1 |  |
|  | PvTGA02 | Phvul.001G249300.1.v2.1 |  |
|  | PvTGA03 | Phvul.003G028800.1.v2.1 |  |
|  | PvTGA04 | Phvul.007G003600.1.v2.1 |  |
|  | PvTGA05 | Phvul.007G025500.1.v2.1 |  |
|  | PvTGA06 | Phvul.008G169000.1.v2.1 |  |
|  | PvTGA07 | Phvul.009G026900.1.v2.1 |  |
|  | PvTGA08 | Phvul.011G203400.1.v2.1 |  |
| *Arachis hypogaea* | AhTGA01 | ArahyR4ID1P |  |
|  | AhTGA02 | ArahyLTZY89 |  |
|  | AhTGA03 | ArahyS1DMKE |  |
|  | AhTGA04 | ArahyRIV6ZB |  |
|  | AhTGA05 | Arahy0GP62G |  |
|  | AhTGA06 | Arahy661MCQ |  |
|  | AhTGA07 | ArahyKZ9VWQ |  |
|  | AhTGA08 | ArahyJ1CFJJ |  |
|  | AhTGA09 | ArahyRKP385 |  |
|  | AhTGA10 | ArahyW15JJQ |  |
|  | AhTGA11 | ArahyP1JBJN |  |
|  | AhTGA12 | Arahy5F9GBV |  |
|  | AhTGA13 | ArahyEH3L7F |  |
|  | AhTGA14 | Arahy6K442L |  |
|  | AhTGA15 | ArahyH6GQLR |  |
|  | AhTGA16 | ArahyPHPT4F |  |
|  | AhTGA17 | Arahy980X4K |  |
|  | AhTGA18 | ArahySERN02 |  |
|  | AhTGA19 | ArahyK04A4E |  |
|  | AhTGA20 | ArahyAAI19J |  |
| *Medicago truncatula* | MtTGA01 | Mt3g044820 |  |
|  | MtTGA02 | Mt3g073560 |  |
|  | MtTGA03 | Mt3g087940 |  |
|  | MtTGA04 | Mt8g040120 |  |
|  | MtTGA05 | Mt7g089800 |  |
|  | MtTGA06 | Mt5g054900 |  |
|  | MtTGA07 | Mt5g060950 |  |
|  | MtTGA08 | Mt1g111320 |  |
|  | MtTGA09 | Mt1g111310 |  |
| *Glycine Max* | GmTGA01 | Glyma01G084200 |  |
|  | GmTGA02 | Glyma02G097900 |  |
|  | GmTGA03 | Glyma02G176800 |  |
|  | GmTGA04 | Glyma03G127600 |  |
|  | GmTGA05 | Glyma03G128200 |  |
|  | GmTGA06 | Glyma03G142400 |  |
|  | GmTGA07 | Glyma04G254800 |  |
|  | GmTGA08 | Glyma05G182500 |  |
|  | GmTGA09 | Glyma06G107300 |  |
|  | GmTGA10 | Glyma08G140100 |  |
|  | GmTGA11 | Glyma10G092100 |  |
|  | GmTGA12 | Glyma10G276100 |  |
|  | GmTGA13 | Glyma10G296200 |  |
|  | GmTGA14 | Glyma11G183700 |  |
|  | GmTGA15 | Glyma11G236300 |  |
|  | GmTGA16 | Glyma12G088700 |  |
|  | GmTGA17 | Glyma12G184500 |  |
|  | GmTGA18 | Glyma13G085100 |  |
|  | GmTGA19 | Glyma13G193700 |  |
|  | GmTGA20 | Glyma13G316900 |  |
|  | GmTGA21 | Glyma14G167000 |  |
|  | GmTGA22 | Glyma15G232000 |  |
|  | GmTGA23 | Glyma18G020900 |  |
|  | GmTGA24 | Glyma19G130200 |  |
|  | GmTGA25 | Glyma19G145300 |  |
|  | GmTGA26 | Glyma20G113600 |  |
|  | GmTGA27 | Glyma20G246400 |  |
| *Sorghum bicolor* | SbTGA01 | Sb010G110100 |  |
|  | SbTGA02 | Sb003G329500 |  |
|  | SbTGA03 | Sb003G081000 |  |
|  | SbTGA04 | Sb009G180700 |  |
| *Oryzae Sativa* | OsbZIP03 | NC_029256.1 |  |
|  | OsbZIP08 | NC_029256.1 |  |
|  | OsbZIP11 | NC_029256.1 |  |
|  | OsbZIP17 | NC_029257.1 |  |
|  | OsbZIP28 | NC_029258.1 |  |
|  | OsbZIP37 | NC_029259.1 |  |
|  | OsbZIP41 | NC_029260.1 |  |
|  | OsbZIP47 | NC_029261.1 |  |
|  | OsbZIP49 | NC_029260.1 |  |
|  | OsbZIP63 | NC_029262.1 |  |
|  | OsbZIP64 | NC_029263.1 |  |
|  | OsbZIP70 | NC_029264.1(LOC4346610 ) |  |
|  | OsbZIP74 | NC_029264.1(LOC4341554) |  |
|  | OsbZIP79 | NC_029266.1 |  |
|  | OsbZIP83 | NC_029267.1 |  |
| *Zea mays* | ZmTGA01 | Zm00001d037317 |  |
|  | ZmTGA02 | Zm00001d043153 |  |
|  | ZmTGA03 | Zm00001d010658 |  |
|  | ZmTGA04 | Zm00001d012553 |  |
|  | ZmTGA05 | Zm00001d008225 |  |
| *Cicer arietinum* | CaTGA01 | Ca09600 |  |
|  | CaTGA02 | Ca11168 |  |
|  | CaTGA03 | Ca17971 |  |
|  | CaTGA04 | Ca25784 |  |
|  | CaTGA05 | Ca10901 |  |
|  | CaTGA06 | Ca05471 |  |
|  | CaTGA07 | Ca07170 |  |
|  | CaTGA08 | Ca12670 |  |
| *Vitis vinifera* | VvTGA01 | VIT_07s0031g01320.t01 |  |
|  | VvTGA02 | VIT_18s0001g04470.t01 |  |
|  | VvTGA03 | VIT_13s0084g00660.t01 |  |
|  | VvTGA04 | VIT_08s0007g05170.t01 |  |
|  | VvTGA05 | VIT_01s0011g03230.t01 |  |
|  | VvTGA06 | VIT_08s0007g06160.t01 |  |
|  | VvTGA07 | VIT_06s0080g00360.t01 |  |

**Table S2:** Collinear gene pairs identified in common bean and other plants

| **Other species** | **Common bean** | **Subgene family clade** |
| --- | --- | --- |
| AhTGA19 | PvTGA03 | Group I |
| AhTGA06 | PvTGA05 | Group I |
| AhTGA19 | PvTGA05 | Group I |
| AhTGA09 | PvTGA08 | Group II |
| AhTGA18 | PvTGA08 | Group II |
| AtTGA8 | PvTGA05 | Group I |
| CaTGA06 | PvTGA05 | Group I |
| CaTGA03 | PvTGA08 | Group II |
| GmTGA01 | PvTGA03 | Group I |
| GmTGA02 | PvTGA03 | Group I |
| GmTGA13 | PvTGA04 | Group II |
| GmTGA27 | PvTGA04 | Group II |
| GmTGA01 | PvTGA05 | Group I |
| GmTGA12 | PvTGA05 | Group I |
| GmTGA26 | PvTGA05 | Group I |
| GmTGA19 | PvTGA08 | Group II |
| GmTGA22 | PvTGA08 | Group II |
| MtTGA09 | PvTGA03 | Group I |
| MtTGA06 | PvTGA03 | Group I |
| MtTGA09 | PvTGA05 | Group I |
| MtTGA04 | PvTGA08 | Group II |
| VvTGA05 | PvTGA03 | Group I |
| VvTGA05 | PvTGA05 | Group I |
| VvTGA03 | PvTGA08 | Group II |

**Table S3:** Ka/Ks value for the collinear gene pairs between common bean and other species

| **Common bean versus Cultivated peanut (*A. hypogaea*)** | | | | |
| --- | --- | --- | --- | --- |
| Gene ID | Gene ID | Ka | Ks | Ka_Ks |
| *PvTGA03* | *AhTGA19* | 0.1953 | 0.8374 | 0.2332 |
| *PvTGA05* | *AhTGA06* | 0.1609 | 0.8738 | 0.1841 |
| *PvTGA05* | *AhTGA19* | 0.1510 | 0.8118 | 0.1860 |
| *PvTGA08* | *AhTGA09* | 0.0392 | 0.5334 | 0.0734 |
| *PvTGA08* | *AhTGA18* | 0.0417 | 0.5520 | 0.0756 |
|  |  |  |  |  |
| **Common bean versus Arabidopsis** | | | | |
| Gene ID | Gene ID | Ka | Ks | Ka_Ks |
| *PvTGA05* | *AtTGA8* | 0.2805 | 2.0964 | 0.1338 |
|  |  |  |  |  |
| **Common bean versus Chickpea** | | | | |
| Gene ID | Gene ID | Ka | Ks | Ka_Ks |
| *PvTGA05* | *CaTGA06* | 0.1195 | 0.7076 | 0.1689 |
| *PvTGA08* | *CaTGA03* | 0.0492 | 0.5525 | 0.0891 |
|  |  |  |  |  |
| **Common bean versus Soybean** | | | | |
| Gene ID | Gene ID | Ka | Ks | Ka_Ks |
| *PvTGA03* | *GmTGA01* | 0.0920 | 0.3074 | 0.2994 |
| *PvTGA03* | *GmTGA02* | 0.0871 | 0.3494 | 0.2493 |
| *PvTGA04* | *GmTGA13* | 0.0185 | 0.2796 | 0.0663 |
| *PvTGA04* | *GmTGA27* | 0.0453 | 0.2567 | 0.1764 |
| *PvTGA05* | *GmTGA01* | 0.1806 | 0.6419 | 0.2813 |
| *PvTGA05* | *GmTGA12* | 0.0573 | 0.3357 | 0.1708 |
| *PvTGA05* | *GmTGA26* | 0.0636 | 0.3036 | 0.2095 |
| *PvTGA08* | *GmTGA19* | 0.0284 | 0.2534 | 0.1122 |
| *PvTGA08* | *GmTGA22* | 0.0731 | 0.3104 | 0.2356 |
|  |  |  |  |  |
| **Common bean versus Medicago truncatula** | | | | |
| Gene ID | Gene ID | Ka | Ks | Ka_Ks |
| *PvTGA03* | *MtTGA09* | 0.2358 | 0.7009 | 0.3364 |
| *PvTGA03* | *MtTGA06* | 0.1568 | 0.6069 | 0.2584 |
| *PvTGA05* | *MtTGA09* | 0.1819 | 0.6568 | 0.2770 |
| *PvTGA08* | *MtTGA04* | 0.0532 | 0.5527 | 0.0962 |
|  |  |  |  |  |
| **Common bean versus Vitis vinifera** | | | | |
| Gene ID | Gene ID | Ka | Ks | Ka_Ks |
| *PvTGA03* | *VvTGA05* | 0.2263 | 1.0652 | 0.2125 |
| *PvTGA05* | *VvTGA05* | 0.1815 | 0.9713 | 0.1868 |
| *PvTGA08* | *VvTGA03* | 0.1352 | 1.0093 | 0.1339 |

**Table S4-1:** *Cis*-regulatory elements in the promoter region of common bean *TGA* in this study

| **Gene** | **Site Name** | **Sequence** | **Position** | **score** | **Strand Matrix** | | **Organism** | **Function** |
| --- | --- | --- | --- | --- | --- | --- | --- | --- |
| *PvTGA01* | G-Box | CACGTT | 351 | 6 | - | Pisum sativum | | cis-acting regulatory element involved in light responsiveness |
| *PvTGA01* | P-box | CCTTTTG | 784 | 7 | + | Oryza sativa | | gibberellin-responsive element |
| *PvTGA01* | ARE | AAACCA | 1644 | 6 | - | Zea mays | | cis-acting regulatory element essential for the anaerobic induction |
| *PvTGA01* | CGTCA-motif | CGTCA | 1053 | 5 | + | Hordeum vulgare | | cis-acting regulatory element involved in the MeJA-responsiveness |
| *PvTGA01* | CGTCA-motif | CGTCA | 1359 | 5 | + | Hordeum vulgare | | cis-acting regulatory element involved in the MeJA-responsiveness |
| *PvTGA01* | AE-box | AGAAACAA | 533 | 8 | - | Arabidopsis thaliana | | part of a module for light response |
| *PvTGA01* | GT1-motif | GGTTAA | 1967 | 6 | + | Arabidopsis thaliana | | light responsive element |
| *PvTGA01* | TGA-element | AACGAC | 1994 | 6 | - | Brassica oleracea | | auxin-responsive element |
| *PvTGA01* | MBS | CAACTG | 530 | 6 | - | Arabidopsis thaliana | | MYB binding site involved in drought-inducibility |
| *PvTGA01* | Box II | TGGTAATAA | 1171 | 9 | + | Solanum tuberosum | | part of a light responsive element |
| *PvTGA01* | GCN4_motif | TGAGTCA | 370 | 7 | + | Oryza sativa | | cis-regulatory element involved in endosperm expression |
| *PvTGA01* | TGACG-motif | TGACG | 1053 | 5 | - | Hordeum vulgare | | cis-acting regulatory element involved in the MeJA-responsiveness |
| *PvTGA01* | TGACG-motif | TGACG | 1359 | 5 | - | Hordeum vulgare | | cis-acting regulatory element involved in the MeJA-responsiveness |
| *PvTGA01* | GARE-motif | TCTGTTG | 764 | 7 | - | Brassica oleracea | | gibberellin-responsive element |
| *PvTGA01* | TCA-element | CCATCTTTTT | 263 | 9 | - | Nicotiana tabacum | | cis-acting element involved in salicylic acid responsiveness |
| *PvTGA01* | TC-rich repeats | ATTCTCTAAC | 399 | 9 | - | Nicotiana tabacum | | cis-acting element involved in defense and stress responsiveness |
| *PvTGA01* | ABRE | ACGTG | 352 | 5 | + | Arabidopsis thaliana | | cis-acting element involved in the abscisic acid responsiveness |
| *PvTGA02* | GT1-motif | GGTTAA | 1827 | 6 | - | Arabidopsis thaliana | | light responsive element |
| *PvTGA02* | Box 4 | ATTAAT | 422 | 6 | + | Petroselinum crispum | | part of a conserved DNA module involved in light responsiveness |
| *PvTGA02* | Box 4 | ATTAAT | 508 | 6 | + | Petroselinum crispum | | part of a conserved DNA module involved in light responsiveness |
| *PvTGA02* | Box 4 | ATTAAT | 1466 | 6 | - | Petroselinum crispum | | part of a conserved DNA module involved in light responsiveness |
| *PvTGA02* | AE-box | AGAAACAA | 119 | 8 | + | Arabidopsis thaliana | | part of a module for light response |
| *PvTGA02* | AE-box | AGAAACAA | 1860 | 8 | - | Arabidopsis thaliana | | part of a module for light response |
| *PvTGA02* | AE-box | AGAAACAA | 1873 | 8 | - | Arabidopsis thaliana | | part of a module for light response |
| *PvTGA02* | MBS | CAACTG | 1517 | 6 | - | Arabidopsis thaliana | | MYB binding site involved in drought-inducibility |
| *PvTGA02* | P-box | CCTTTTG | 1755 | 7 | + | Oryza sativa | | gibberellin-responsive element |
| *PvTGA02* | G-Box | CACGTT | 1530 | 6 | - | Pisum sativum | | cis-acting regulatory element involved in light responsiveness |
| *PvTGA02* | O2-site | GATGATGTGG | 1536 | 9 | + | Zea mays | | cis-acting regulatory element involved in zein metabolism regulation |
| *PvTGA02* | GATA-motif | GATAGGA | 1013 | 7 | + | Arabidopsis thaliana | | part of a light responsive element |
| *PvTGA02* | CGTCA-motif | CGTCA | 382 | 5 | - | Hordeum vulgare | | cis-acting regulatory element involved in the MeJA-responsiveness |
| *PvTGA02* | CGTCA-motif | CGTCA | 703 | 5 | - | Hordeum vulgare | | cis-acting regulatory element involved in the MeJA-responsiveness |
| *PvTGA02* | ARE | AAACCA | 28 | 6 | - | Zea mays | | cis-acting regulatory element essential for the anaerobic induction |
| *PvTGA02* | ARE | AAACCA | 1136 | 6 | - | Zea mays | | cis-acting regulatory element essential for the anaerobic induction |
| *PvTGA02* | ARE | AAACCA | 1415 | 6 | - | Zea mays | | cis-acting regulatory element essential for the anaerobic induction |
| *PvTGA02* | TC-rich repeats | ATTCTCTAAC | 829 | 9 | + | Nicotiana tabacum | | cis-acting element involved in defense and stress responsiveness |
| *PvTGA02* | ABRE | GACACGTGGC | 716 | 9 | + | Triticum aestivum | | cis-acting element involved in the abscisic acid responsiveness |
| *PvTGA02* | ABRE | ACGTG | 1531 | 5 | + | Arabidopsis thaliana | | cis-acting element involved in the abscisic acid responsiveness |
| *PvTGA02* | MRE | AACCTAA | 352 | 7 | - | Petroselinum crispum | | MYB binding site involved in light responsiveness |
| *PvTGA02* | TGACG-motif | TGACG | 382 | 5 | + | Hordeum vulgare | | cis-acting regulatory element involved in the MeJA-responsiveness |
| *PvTGA02* | TGACG-motif | TGACG | 703 | 5 | + | Hordeum vulgare | | cis-acting regulatory element involved in the MeJA-responsiveness |
| *PvTGA02* | I-box | gGATAAGGTG | 1535 | 9 | + | Zea mays | | part of a light responsive element |
| *PvTGA02* | TCA-element | TCAGAAGAGG | 981 | 9 | - | Brassica oleracea | | cis-acting element involved in salicylic acid responsiveness |
| *PvTGA02* | 3-AF1 binding site | TAAGAGAGGAA | 322 | 10 | + | Solanum tuberosum | | light responsive element |
| *PvTGA02* | TCT-motif | TCTTAC | 321 | 6 | - | Arabidopsis thaliana | | part of a light responsive element |
| *PvTGA03* | chs-CMA1a | TTACTTAA | 831 | 8 | - | Daucus carota | | part of a light responsive element |
| *PvTGA03* | G-box | TACGTG | 1593 | 6 | - | Arabidopsis thaliana | | cis-acting regulatory element involved in light responsiveness |
| *PvTGA03* | AuxRE | TGTCTCAATAAG | 1857 | 11 | - | Glycine max | | part of an auxin-responsive element |
| *PvTGA03* | CGTCA-motif | CGTCA | 38 | 5 | - | Hordeum vulgare | | cis-acting regulatory element involved in the MeJA-responsiveness |
| *PvTGA03* | CGTCA-motif | CGTCA | 1970 | 5 | - | Hordeum vulgare | | cis-acting regulatory element involved in the MeJA-responsiveness |
| *PvTGA03* | GATA-motif | AAGATAAGATT | 634 | 10 | - | Arabidopsis thaliana | | part of a light responsive element |
| *PvTGA03* | GATA-motif | AAGATAAGATT | 930 | 10 | - | Arabidopsis thaliana | | part of a light responsive element |
| *PvTGA03* | ARE | AAACCA | 419 | 6 | + | Zea mays | | cis-acting regulatory element essential for the anaerobic induction |
| *PvTGA03* | GT1-motif | GGTTAAT | 861 | 7 | - | Avena sativa | | light responsive element |
| *PvTGA03* | GT1-motif | GGTTAA | 862 | 6 | - | Arabidopsis thaliana | | light responsive element |
| *PvTGA03* | GT1-motif | GGTTAA | 1467 | 6 | - | Arabidopsis thaliana | | light responsive element |
| *PvTGA03* | AE-box | AGAAACAA | 1977 | 8 | + | Arabidopsis thaliana | | part of a module for light response |
| *PvTGA03* | Box 4 | ATTAAT | 943 | 6 | + | Petroselinum crispum | | part of a conserved DNA module involved in light responsiveness |
| *PvTGA03* | TCCC-motif | TCTCCCT | 796 | 7 | + | Spinacia oleracea | | part of a light responsive element |
| *PvTGA03* | AT-rich element | ATAGAAATCAA | 944 | 10 | - | Glycine max | | binding site of AT-rich DNA binding protein (ATBP-1) |
| *PvTGA03* | TGACG-motif | TGACG | 38 | 5 | + | Hordeum vulgare | | cis-acting regulatory element involved in the MeJA-responsiveness |
| *PvTGA03* | TGACG-motif | TGACG | 1970 | 5 | + | Hordeum vulgare | | cis-acting regulatory element involved in the MeJA-responsiveness |
| *PvTGA03* | I-box | cCATATCCAAT | 499 | 10 | - | Flaveria trinervia | | part of a light responsive element |
| *PvTGA03* | I-box | gGATAAGGTG | 1121 | 9 | - | Zea mays | | part of a light responsive element |
| *PvTGA03* | I-box | AGATAAGG | 1123 | 8 | - | Triticum aestivum | | part of a light responsive element |
| *PvTGA03* | TCT-motif | TCTTAC | 776 | 6 | + | Arabidopsis thaliana | | part of a light responsive element |
| *PvTGA03* | TCA-element | TCAGAAGAGG | 190 | 9 | - | Brassica oleracea | | cis-acting element involved in salicylic acid responsiveness |
| *PvTGA03* | TCA-element | TCAGAAGAGG | 1851 | 9 | - | Brassica oleracea | | cis-acting element involved in salicylic acid responsiveness |
| *PvTGA03* | TC-rich repeats | GTTTTCTTAC | 884 | 9 | + | Nicotiana tabacum | | cis-acting element involved in defense and stress responsiveness |
| *PvTGA03* | ABRE | CGCACGTGTC | 958 | 9 | + | Hordeum vulgare | | cis-acting element involved in the abscisic acid responsiveness |
| *PvTGA03* | ABRE | ACGTG | 1593 | 5 | - | Arabidopsis thaliana | | cis-acting element involved in the abscisic acid responsiveness |
| *PvTGA03* | MRE | AACCTAA | 318 | 7 | - | Petroselinum crispum | | MYB binding site involved in light responsiveness |
| *PvTGA04* | GARE-motif | TCTGTTG | 1318 | 7 | - | Brassica oleracea | | gibberellin-responsive element |
| *PvTGA04* | CCAAT-box | CAACGG | 1688 | 6 | + | Hordeum vulgare | | MYBHv1 binding site |
| *PvTGA04* | TCT-motif | TCTTAC | 1046 | 6 | - | Arabidopsis thaliana | | part of a light responsive element |
| *PvTGA04* | TCT-motif | TCTTAC | 1077 | 6 | - | Arabidopsis thaliana | | part of a light responsive element |
| *PvTGA04* | TCT-motif | TCTTAC | 1723 | 6 | - | Arabidopsis thaliana | | part of a light responsive element |
| *PvTGA04* | ATCT-motif | AATCTAATCC | 1465 | 9 | - | Pisum sativum | | part of a conserved DNA module involved in light responsiveness |
| *PvTGA04* | ARE | AAACCA | 1493 | 6 | - | Zea mays | | cis-acting regulatory element essential for the anaerobic induction |
| *PvTGA04* | ARE | AAACCA | 1643 | 6 | - | Zea mays | | cis-acting regulatory element essential for the anaerobic induction |
| *PvTGA04* | P-box | CCTTTTG | 1853 | 7 | + | Oryza sativa | | gibberellin-responsive element |
| *PvTGA04* | GA-motif | ATAGATAA | 896 | 8 | + | Arabidopsis thaliana | | part of a light responsive element |
| *PvTGA04* | O2-site | GATGATGTGG | 1356 | 10 | - | Zea mays | | cis-acting regulatory element involved in zein metabolism regulation |
| *PvTGA04* | TCCC-motif | TCTCCCT | 704 | 7 | + | Spinacia oleracea | | part of a light responsive element |
| *PvTGA04* | MBS | CAACTG | 1669 | 6 | - | Arabidopsis thaliana | | MYB binding site involved in drought-inducibility |
| *PvTGA04* | GT1-motif | GGTTAA | 688 | 6 | + | Arabidopsis thaliana | | light responsive element |
| *PvTGA04* | Box 4 | ATTAAT | 1151 | 6 | - | Petroselinum crispum | | part of a conserved DNA module involved in light responsiveness |
| *PvTGA04* | GC-motif | CCCCCG | 1509 | 6 | - | Zea mays | | enhancer-like element involved in anoxic specific inducibility |
| *PvTGA05* | MRE | AACCTAA | 168 | 7 | + | Petroselinum crispum | | MYB binding site involved in light responsiveness |
| *PvTGA05* | MRE | AACCTAA | 225 | 7 | + | Petroselinum crispum | | MYB binding site involved in light responsiveness |
| *PvTGA05* | MRE | AACCTAA | 295 | 7 | + | Petroselinum crispum | | MYB binding site involved in light responsiveness |
| *PvTGA05* | ABRE | ACGTG | 934 | 5 | - | Arabidopsis thaliana | | cis-acting element involved in the abscisic acid responsiveness |
| *PvTGA05* | 3-AF1 binding site | TAAGAGAGGAA | 1099 | 10 | + | Solanum tuberosum | | light responsive element |
| *PvTGA05* | I-box | ccttatcct | 625 | 9 | - | Arabidopsis thaliana | | part of a light responsive element |
| *PvTGA05* | TGACG-motif | TGACG | 936 | 5 | - | Hordeum vulgare | | cis-acting regulatory element involved in the MeJA-responsiveness |
| *PvTGA05* | MBS | CAACTG | 1916 | 6 | + | Arabidopsis thaliana | | MYB binding site involved in drought-inducibility |
| *PvTGA05* | Box 4 | ATTAAT | 638 | 6 | + | Petroselinum crispum | | part of a conserved DNA module involved in light responsiveness |
| *PvTGA05* | Box 4 | ATTAAT | 1004 | 6 | + | Petroselinum crispum | | part of a conserved DNA module involved in light responsiveness |
| *PvTGA05* | GT1-motif | GGTTAAT | 243 | 7 | - | Avena sativa | | light responsive element |
| *PvTGA05* | GT1-motif | GGTTAA | 244 | 6 | - | Arabidopsis thaliana | | light responsive element |
| *PvTGA05* | GT1-motif | GGTTAA | 522 | 6 | - | Arabidopsis thaliana | | light responsive element |
| *PvTGA05* | GT1-motif | GGTTAAT | 1012 | 7 | - | Avena sativa | | light responsive element |
| *PvTGA05* | GT1-motif | GGTTAA | 1013 | 6 | - | Arabidopsis thaliana | | light responsive element |
| *PvTGA05* | ARE | AAACCA | 577 | 6 | + | Zea mays | | cis-acting regulatory element essential for the anaerobic induction |
| *PvTGA05* | ARE | AAACCA | 589 | 6 | + | Zea mays | | cis-acting regulatory element essential for the anaerobic induction |
| *PvTGA05* | ARE | AAACCA | 614 | 6 | - | Zea mays | | cis-acting regulatory element essential for the anaerobic induction |
| *PvTGA05* | GATA-motif | AAGGATAAGG | 624 | 10 | + | Solanum tuberosum | | part of a light responsive element |
| *PvTGA05* | CGTCA-motif | CGTCA | 936 | 5 | + | Hordeum vulgare | | cis-acting regulatory element involved in the MeJA-responsiveness |
| *PvTGA05* | G-box | CACGTC | 934 | 6 | + | Zea mays | | cis-acting regulatory element involved in light responsiveness |
| *PvTGA05* | P-box | CCTTTTG | 449 | 7 | - | Oryza sativa | | gibberellin-responsive element |
| *PvTGA06* | chs-CMA1a | TTACTTAA | 1162 | 8 | + | Daucus carota | | part of a light responsive element |
| *PvTGA06* | P-box | CCTTTTG | 1997 | 7 | + | Oryza sativa | | gibberellin-responsive element |
| *PvTGA06* | ARE | AAACCA | 1776 | 6 | - | Zea mays | | cis-acting regulatory element essential for the anaerobic induction |
| *PvTGA06* | ARE | AAACCA | 1828 | 6 | + | Zea mays | | cis-acting regulatory element essential for the anaerobic induction |
| *PvTGA06* | TATC-box | TATCCCA | 1484 | 7 | - | Oryza sativa | | cis-acting element involved in gibberellin-responsiveness |
| *PvTGA06* | AE-box | AGAAACAA | 482 | 8 | + | Arabidopsis thaliana | | part of a module for light response |
| *PvTGA06* | Box 4 | ATTAAT | 311 | 6 | + | Petroselinum crispum | | part of a conserved DNA module involved in light responsiveness |
| *PvTGA06* | Box 4 | ATTAAT | 839 | 6 | + | Petroselinum crispum | | part of a conserved DNA module involved in light responsiveness |
| *PvTGA06* | Box 4 | ATTAAT | 1652 | 6 | - | Petroselinum crispum | | part of a conserved DNA module involved in light responsiveness |
| *PvTGA06* | MBS | CAACTG | 910 | 6 | + | Arabidopsis thaliana | | MYB binding site involved in drought-inducibility |
| *PvTGA06* | TCT-motif | TCTTAC | 1067 | 6 | + | Arabidopsis thaliana | | part of a light responsive element |
| *PvTGA06* | TCA-element | CCATCTTTTT | 1675 | 9 | + | Nicotiana tabacum | | cis-acting element involved in salicylic acid responsiveness |
| *PvTGA06* | I-box | atGATAAGGTC | 166 | 11 | + | Helianthus annuus | | part of a light responsive element |
| *PvTGA06* | TC-rich repeats | GTTTTCTTAC | 1063 | 9 | + | Nicotiana tabacum | | cis-acting element involved in defense and stress responsiveness |
| *PvTGA06* | MRE | AACCTAA | 158 | 7 | + | Petroselinum crispum | | MYB binding site involved in light responsiveness |
| *PvTGA07* | ARE | AAACCA | 481 | 6 | + | Zea mays | | cis-acting regulatory element essential for the anaerobic induction |
| *PvTGA07* | ARE | AAACCA | 963 | 6 | + | Zea mays | | cis-acting regulatory element essential for the anaerobic induction |
| *PvTGA07* | CGTCA-motif | CGTCA | 1584 | 5 | - | Hordeum vulgare | | cis-acting regulatory element involved in the MeJA-responsiveness |
| *PvTGA07* | CGTCA-motif | CGTCA | 1615 | 5 | - | Hordeum vulgare | | cis-acting regulatory element involved in the MeJA-responsiveness |
| *PvTGA07* | CGTCA-motif | CGTCA | 1932 | 5 | + | Hordeum vulgare | | cis-acting regulatory element involved in the MeJA-responsiveness |
| *PvTGA07* | P-box | CCTTTTG | 1659 | 7 | + | Oryza sativa | | gibberellin-responsive element |
| *PvTGA07* | G-box | TACGTG | 72 | 6 | - | Arabidopsis thaliana | | cis-acting regulatory element involved in light responsiveness |
| *PvTGA07* | G-box | CACGAC | 94 | 6 | + | Zea mays | | cis-acting regulatory element involved in light responsiveness |
| *PvTGA07* | G-box | TACGTG | 794 | 6 | - | Arabidopsis thaliana | | cis-acting regulatory element involved in light responsiveness |
| *PvTGA07* | TGA-element | AACGAC | 1487 | 6 | - | Brassica oleracea | | auxin-responsive element |
| *PvTGA07* | MBS | CAACTG | 1290 | 6 | + | Arabidopsis thaliana | | MYB binding site involved in drought-inducibility |
| *PvTGA07* | Box 4 | ATTAAT | 1280 | 6 | - | Petroselinum crispum | | part of a conserved DNA module involved in light responsiveness |
| *PvTGA07* | TCT-motif | TCTTAC | 104 | 6 | - | Arabidopsis thaliana | | part of a light responsive element |
| *PvTGA07* | TCT-motif | TCTTAC | 343 | 6 | + | Arabidopsis thaliana | | part of a light responsive element |
| *PvTGA07* | TGACG-motif | TGACG | 1584 | 5 | + | Hordeum vulgare | | cis-acting regulatory element involved in the MeJA-responsiveness |
| *PvTGA07* | TGACG-motif | TGACG | 1615 | 5 | + | Hordeum vulgare | | cis-acting regulatory element involved in the MeJA-responsiveness |
| *PvTGA07* | TGACG-motif | TGACG | 1932 | 5 | - | Hordeum vulgare | | cis-acting regulatory element involved in the MeJA-responsiveness |
| *PvTGA07* | ABRE | ACGTG | 72 | 5 | - | Arabidopsis thaliana | | cis-acting element involved in the abscisic acid responsiveness |
| *PvTGA07* | ABRE | ACGTG | 794 | 5 | - | Arabidopsis thaliana | | cis-acting element involved in the abscisic acid responsiveness |
| *PvTGA08* | TCT-motif | TCTTAC | 486 | 6 | - | Arabidopsis thaliana | | part of a light responsive element |
| *PvTGA08* | TCA-element | CCATCTTTTT | 950 | 9 | + | Nicotiana tabacum | | cis-acting element involved in salicylic acid responsiveness |
| *PvTGA08* | Sp1 | GGGCGG | 1593 | 6 | + | Oryza sativa | | light responsive element |
| *PvTGA08* | TC-rich repeats | ATTCTCTAAC | 40 | 9 | + | Nicotiana tabacum | | cis-acting element involved in defense and stress responsiveness |
| *PvTGA08* | MRE | AACCTAA | 499 | 7 | - | Petroselinum crispum | | MYB binding site involved in light responsiveness |
| *PvTGA08* | MRE | AACCTAA | 1958 | 7 | - | Petroselinum crispum | | MYB binding site involved in light responsiveness |
| *PvTGA08* | chs-CMA1a | TTACTTAA | 482 | 8 | - | Daucus carota | | part of a light responsive element |
| *PvTGA08* | chs-CMA1a | TTACTTAA | 1237 | 8 | + | Daucus carota | | part of a light responsive element |
| *PvTGA08* | G-box | CAGACGTGGCA | 1864 | 10 | - | Nicotiana plumbaginifolia | | cis-acting regulatory element involved in light responsiveness |
| *PvTGA08* | LTR | CCGAAA | 1579 | 6 | - | Hordeum vulgare | | cis-acting element involved in low-temperature responsiveness |
| *PvTGA08* | O2-site | GATGATGTGG | 1994 | 9 | + | Zea mays | | cis-acting regulatory element involved in zein metabolism regulation |
| *PvTGA08* | ARE | AAACCA | 1555 | 6 | - | Zea mays | | cis-acting regulatory element essential for the anaerobic induction |
| *PvTGA08* | ARE | AAACCA | 1776 | 6 | - | Zea mays | | cis-acting regulatory element essential for the anaerobic induction |
| *PvTGA08* | ARE | AAACCA | 1823 | 6 | - | Zea mays | | cis-acting regulatory element essential for the anaerobic induction |
| *PvTGA08* | ARE | AAACCA | 1891 | 6 | - | Zea mays | | cis-acting regulatory element essential for the anaerobic induction |
| *PvTGA08* | AT1-motif | AATTATTTTTTATT | 1471 | 13 | + | Solanum tuberosum | | part of a light responsive module |
| *PvTGA08* | GT1-motif | GTGTGTGAA | 1638 | 9 | - | Solanum tuberosum | | light responsive element |
| *PvTGA08* | AE-box | AGAAACAA | 1839 | 8 | - | Arabidopsis thaliana | | part of a module for light response |
| *PvTGA08* | Box 4 | ATTAAT | 814 | 6 | + | Petroselinum crispum | | part of a conserved DNA module involved in light responsiveness |
| *PvTGA08* | Box 4 | ATTAAT | 1032 | 6 | - | Petroselinum crispum | | part of a conserved DNA module involved in light responsiveness |
| *PvTGA08* | MBS | CAACTG | 1388 | 6 | + | Arabidopsis thaliana | | MYB binding site involved in drought-inducibility |

**Table S4-2:** *Cis-*regulatory elements number in the promoter region of common bean in this study

| **Gene.ID** | **ARE** | **Box 4** | **GT1-motif** | **3-AF1 binding site** | **Sp1** | **TCT-motif** | **MRE** | **I-box** | **G-Box** | **AE-box** | **AT1-motif** | **TCCC-motif** | **AuxRE** | **ATCT-motif** | **chs-CMA1a** | **GATA-motif** | **O2-site** | **Box II** |
| --- | --- | --- | --- | --- | --- | --- | --- | --- | --- | --- | --- | --- | --- | --- | --- | --- | --- | --- |
| *PvTGA01* | 1 | 0 | 1 | 0 | 0 | 0 | 0 | 0 | 1 | 1 | 0 | 0 | 0 | 0 | 0 | 0 | 0 | 1 |
| *PvTGA02* | 3 | 3 | 1 | 1 | 0 | 1 | 1 | 1 | 1 | 3 | 0 | 0 | 0 | 0 | 0 | 1 | 1 | 0 |
| *PvTGA03* | 1 | 1 | 3 | 0 | 0 | 1 | 1 | 3 | 1 | 1 | 0 | 1 | 1 | 0 | 1 | 2 | 0 | 0 |
| *PvTGA04* | 2 | 1 | 1 | 0 | 0 | 3 | 0 | 0 | 0 | 0 | 0 | 1 | 0 | 1 | 0 | 0 | 1 | 0 |
| *PvTGA05* | 3 | 2 | 5 | 1 | 0 | 0 | 3 | 1 | 1 | 0 | 0 | 0 | 0 | 0 | 0 | 1 | 0 | 0 |
| *PvTGA06* | 2 | 3 | 0 | 0 | 0 | 1 | 1 | 1 | 0 | 1 | 0 | 0 | 0 | 0 | 1 | 0 | 0 | 0 |
| *PvTGA07* | 2 | 1 | 0 | 0 | 0 | 2 | 0 | 0 | 3 | 0 | 0 | 0 | 0 | 0 | 0 | 0 | 0 | 0 |
| *PvTGA08* | 4 | 2 | 1 | 0 | 1 | 1 | 2 | 0 | 1 | 1 | 1 | 0 | 0 | 0 | 2 | 0 | 1 | 0 |

| **Gene.ID** | **GCN4_motif** | **AT-rich element** | **GA-motif** | **GC-motif** | **TC-rich repeats** | **MBS** | **LTR** | **CCAAT-box** | **GARE-motif** | **TGA-element** | **P-box** | **TCA-element** | **ABRE** | **TGACG-motif** | **CGTCA-motif** | **TATC-box** | |
| --- | --- | --- | --- | --- | --- | --- | --- | --- | --- | --- | --- | --- | --- | --- | --- | --- | --- |
| *PvTGA01* | 1 | 0 | 0 | 0 | 1 | 1 | 0 | 0 | 1 | 1 | 1 | 1 | 1 | 2 | 2 | 0 |  |
| *PvTGA02* | 0 | 0 | 0 | 0 | 1 | 1 | 0 | 0 | 0 | 0 | 1 | 1 | 2 | 2 | 2 | 0 |  |
| *PvTGA03* | 0 | 1 | 0 | 0 | 1 | 0 | 0 | 0 | 0 | 0 | 0 | 2 | 2 | 2 | 2 | 0 |  |
| *PvTGA04* | 0 | 0 | 1 | 1 | 0 | 1 | 0 | 1 | 1 | 0 | 1 | 0 | 0 | 0 | 0 | 0 |  |
| *PvTGA05* | 0 | 0 | 0 | 0 | 0 | 1 | 0 | 0 | 0 | 0 | 1 | 0 | 1 | 1 | 1 | 0 |  |
| *PvTGA06* | 0 | 0 | 0 | 0 | 1 | 1 | 0 | 0 | 0 | 0 | 1 | 1 | 0 | 0 | 0 | 1 |  |
| *PvTGA07* | 0 | 0 | 0 | 0 | 0 | 1 | 0 | 0 | 0 | 1 | 1 | 0 | 2 | 3 | 3 | 0 |  |
| *PvTGA08* | 0 | 0 | 0 | 0 | 1 | 1 | 1 | 0 | 0 | 0 | 0 | 1 | 0 | 0 | 0 | 0 |  |

**Table S4-3:** Classification criteria for *Cis-*regulatory elements in promoters

| **Founctions ^Group^** | **plant growth and development** |
| --- | --- |
| anaerobic induction | ARE |
| light responsive | Box 4 |
|  | GT1-motif |
|  | 3-AF1 binding site |
|  | Sp1 |
|  | TCT-motif |
|  | MRE |
|  | I-box |
|  | G-Box |
|  | AE-box |
|  | AT1-motif |
|  | TCCC-motif |
|  | ATCT-motif |
|  | chs-CMA1a |
|  | GATA-motif |
|  | Box II |
|  | AT-rich element |
|  | GA-motif |
| endosperm expression | GCN4_motif |
| anoxic specific inducibility | GC-motif |
| auxin-responsive | AuxRE |
| zein metabolism regulation | O2-site |

| **Founctions ^Group^** | **stree responsive** |
| --- | --- |
| defense and stress responsiveness | TC-rich repeats |
| drought-inducibility | MBS |
|  | CCAAT-box |
| low-temperature responsive | LTR |

| **Founctions ^Group^** | **hormone responsive** |
| --- | --- |
| gibberellin-responsive | GARE-motif |
|  | P-box |
|  | TATC-box |
| abscisic acid responsive | ABRE |
| auxin-responsive | TGA-element |
| salicylic acid responsive | TCA-element |
| MeJA-responsive | TGACG-motif |
|  | CGTCA-motif |

**Table S5:** Primers of the candidate genes for real-time PCR

| **Gene name** | **Primer sequence (5'-3')** | |
| --- | --- | --- |
| *PvTGA01* | forward primer | TGCCATTCGTTAGGGTTTGC |
|  | reverse primer | CCCTGGTCGCATGAAAATCG |
| *PvTGA02* | forward primer | TGTAGATCAACACAAGCAAGCA |
|  | reverse primer | TGCAGAGACGACTTTTTGGTG |
| *PvTGA03* | forward primer | TTCTGCTATCTGAACACAACAACA |
|  | reverse primer | GGGACCGACCAGACCAAAAA |
| *PvTGA04* | forward primer | CTCTTCCTTTCACTTTAATCCAACA |
|  | reverse primer | TGCAGCCCAACAAAATTCAAGA |
| *PvTGA05* | forward primer | TGCTTCCAACTGCATTTCCAT |
|  | reverse primer | CGGTTGGGTTGAAGCTTTGC |
| *PvTGA06* | forward primer | GGTGCTTGCAGATTCTACCCA |
|  | reverse primer | CGCGAGGACTAGTTGAGTCG |
| *PvTGA07* | forward primer | CTGGGAGATCTTCGACTAACCA |
|  | reverse primer | AGCTCTCAACCTCTAACCAGA |
| *PvTGA08* | forward primer | AGCAGAAGAAGACATCCAGAGA |
|  | reverse primer | ACTGTTCAACCTTGTATGTAGTT |
